# Uptake and determinants of immediate and extended postpartum long-acting reversible contraceptive use in Eastern and Western Africa: A systematic review and meta-analysis

**DOI:** 10.1371/journal.pone.0346885

**Published:** 2026-04-17

**Authors:** Ritbano Ahmed Abdo, Endalew Gemechu Sendo, Erdaw Tachbele Betre, Kedir Hussen

**Affiliations:** 1 Department of Midwifery, Wachemo University, College of Medicine and Health Sciences, Hossana, Ethiopia; 2 Addis Ababa University, College of Health Sciences, School of Nursing and Midwifery, Addis Ababa, Ethiopia; 3 Department of Nursing, Worabe University, College of Health Sciences, Worabe, Ethiopia; Universidad Peruana Cayetano Heredia Facultad de Medicina, PERU

## Abstract

**Background:**

Reducing unintended pregnancies and improving maternal/child health relies heavily on postpartum family planning (PPFP). Despite their high effectiveness, the use of long-acting reversible contraceptives (LARC), such as intrauterine contraceptive device (IUCD) and implants, is inconsistent in Africa after childbirth. This systematic review and meta-analysis aimed to assess the prevalence and determinants of immediate and extended postpartum use of long-acting reversible contraceptives in Eastern and Western Africa.

**Methods:**

We searched PubMed, the Cochrane Library, Hinari, and gray literature sources (Google Scholar) for studies published between January 1, 2015, and December 12, 2025. Eligible studies reported LARC uptake within 12 months of postpartum. Heterogeneity was assessed using the I² statistic, publication bias using Egger’s test, and study quality using the JBI 2020 checklist. Data were analyzed using Stata version 17 and R version 4.5.

**Results:**

Sixty-eight studies were included, most of which were from Eastern Africa. The pooled prevalence was 21.7% for immediate postpartum LARC (IPP-LARC) and 19.5% for extended postpartum LARC (EPP-LARC). Uptake was significantly associated with counseling during maternal and child health visits, partner involvement, prior family planning use, and good knowledge. Age ≥ 35 years and higher education level also favored immediate postpartum IUD use.

**Conclusion:**

The uptake of postpartum LARCs remains low in Eastern and Western Africa. Strengthening FP counseling during MCH services, encouraging partner involvement, and improving knowledge are critical strategies to increase coverage and reduce unmet needs.

**Trial registration:**

PROSPERO International Prospective Register of Systematic Reviews (ID: CRD420250577642).

## Introduction

Maternal health conditions in Sub-Saharan Africa (SSA) remain a principal global health challenge, characterized by persistently high rates of maternal mortality and morbidity [1–3]. This public health crisis is critically fueled by unintended [4–6] and closely spaced pregnancies [7,8], which are potent risk factors for a series of adverse outcomes, including unsafe abortion, preterm delivery, and low infant birth weight [7,9–13]. The persistence of these conditions underscores profound deficiencies in reproductive healthcare systems and highlights the urgent need to strengthen contraceptive access and utilization as a foundational strategy for improving maternal survival.

The postpartum period is a particularly strategic interval for interventions [14,15]. During this time, nearly one-fourth of women in SSA experienced an unmet need for family planning, desiring to avoid pregnancy while not using contraception [16–18]. This gap represents a critical failure in service delivery, often described as a “missed opportunity” for facility-based childbirth. Despite direct contact with the healthcare system, many women are discharged without integrated counseling or contraceptive provision [19,20], perpetuating rapid, high-risk repeat pregnancies [19,21–26].

Long-acting reversible contraceptives (LARCs), including implants and intrauterine contraceptive device (IUCD) [27–31], are highly effective, safe for most women, and provide long-term, user-independent protection [32–35]. Their “forgettable” nature is especially advantageous in meeting the demands of the postpartum and infant-care periods [35–37]. Immediate postpartum initiation (within 48 h of delivery) leverages high motivation and existing clinical infrastructure [17], whereas extended postpartum use (up to 12 months) ensures sustained protection and supports the WHO-recommended birth-to-pregnancy interval of at least 24 months [38].

Despite the recognized potential of LARC, evidence on postpartum uptake across Africa remains fragmented [21,22,39–44]. Studies vary in methodology, regional focus, and definitions of postpartum timing, limiting a consolidated understanding of prevalence, determinants, and regional differences.

Therefore, this systematic review and meta-analysis synthesizes the evidence on uptake and determinants of immediate and extended postpartum LARC use in Eastern and Western Africa. By separately pooling prevalence estimates for timing and method type, this review provides nuanced insights to guide policy, resource allocation, and clinical practice aimed at reducing maternal and infant mortality and advancing reproductive autonomy across the continent.

## Methods

### Study protocol and reporting

This systematic review adhered to the Preferred Reporting Items for Systematic Reviews and Meta-Analyses (PRISMA) 2020 guidelines [45]. The study protocol, developed in accordance with these standards, was registered with PROSPERO (CRD420250577642). The eligibility criteria were formulated based on the Joanna Briggs Institute (JBI) 2017 review guidelines [46]. For the review process, Rayyan (a specialised, AI-powered systematic review management platform) was used to facilitate the screening process. This included the removal of duplicates (manual) and the blind screening of titles and abstracts by two independent reviewers to minimise selection bias [47]. Furthermore, EndNote (version 21) was employed for managing and citing related articles throughout the study [48].

### Inclusion criteria

**Study area**: Eastern and Western Africa. **Study participants**: The postpartum women within 12 months of delivery, regardless of their age, parity, or marital status were considered. **Types of studies***:* Cross-sectional study, case–control and mixed-methods studies were considered. Although some studies evaluated service delivery strategies, such as quality improvement cycles or provider counseling initiatives, those employing pre–post observational designs were classified as observational, and only baseline prevalence data were extracted and synthesized in the meta-analysis. This decision was made to preserve methodological consistency and ensure comparability across the pooled prevalence estimates.

**Outcome of interests**: The primary focus of this review was to examine the prevalence of postpartum long-acting contraceptive uptake (both immediate and extended period) and its associated factors.

**Publication condition**: Both published and unpublished literature were considered, including peer-reviewed- journal articles, theses, and official institutional reports. **Language**: Any language was considered but all articles identified during database searches were English language. **Publication date***:* To ensure relevance to contemporary contraceptive practices, only studies published between January 1, 2015, and October 12, 2025, were included.

### Exclusion criteria

Studies were excluded if they employed purely qualitative methodologies or were non-research publications, such as editorials, commentaries, and narrative reviews. Studies that focused exclusively on non-LARC methods or failed to disaggregate LARC outcomes from other contraceptive data were also excluded. Conference abstracts lacking sufficient methodological details for appraisal or data extraction were excluded. Duplicate studies using the same data source were removed, retaining only the versions with the highest methodological quality scores. Similarly, duplicate publications of the same study were excluded, retaining only the most comprehensive version. Finally, records for which the full text could not be accessed after exhaustive retrieval efforts were excluded from the study.

### Measurement

#### Primary outcome.

The primary outcome of interest was the pooled prevalence of LARC use among postpartum women in the included studies. For analytical clarity, prevalence estimates were stratified into two main categories: IPP-LARC and EPP-LARC. Each category was further disaggregated by method type, yielding four subgroups: immediate postpartum implant (IPP-I), immediate postpartum IUD (IPP-IUCD), extended postpartum implant (EPP-I), and extended postpartum IUCD (EPP-IUCD).

1. **IPPL-ARC:** Uptake of either IUCD or implants within 48 h of delivery [33]**.**
2. **EPP-LARC:** Uptake of IUCD or implants initiated after 48 hours to 12 months following childbirth (i.e., within the first year postpartum) [49,50].

#### Secondary outcomes.

The secondary outcomes included factors influencing the uptake of IPP-LARCs. In the meta-analysis-, family planning counseling was considered present when studies reported that women received counseling during the continuum of maternity care, including antenatal care (ANC) contacts, intrapartum, or the immediate postpartum period, although most data were derived from ANC visits. Partner involvement was defined as male participation in family planning through support, attendance at counseling sessions, or discussions between the mother and her partner. The participants’ knowledge was assessed based on their understanding of IPP-LARC and categorized as either good or poor, while attitude was classified as positive or negative toward its utilization. Previous modern contraceptive exposure was defined as the prior uptake of LARC or the use of any modern contraceptive method. The mode of delivery was analyzed by comparing normal spontaneous vaginal delivery (NSVD) with cesarean delivery. Finally, the desire for future children was defined as whether women reported wanting another child in the future.

### Information sources and search strategy

We searched PubMed, Hinari, Cochrane, Google Scholar, and ProQuest Dissertations and Theses databases to identify relevant studies. The search strategy was developed in three phases to ensure sensitivity and precision of the search. First, an exploratory search in PubMed and Google Scholar was performed to identify relevant keywords and controlled vocabulary terms (e.g., Medical Subject Headings). These terms were then combined to construct a full search strategy, which was adapted to the syntax and subject headings of each database. Core concepts included the Immediate postpartum, “Extended postpartum period,” “Long-acting contracept*” LARCs, Intrauterine device, Intrauterine contraceptive device, uptake, utilization, use, adoption, determinants, associated factors, women, mothers, and Africa. For example, the PubMed strategy combined terms such as (“Postpartum Period” [Mesh] OR “postpartum”) AND (“Long-Acting Reversible Contraception” [Mesh] OR “LARC*”) AND (“Immediate postpartum” OR “Extended postpartum period”) AND (“Africa” [Mesh] OR “Africa”). Finally, supplementary searches were conducted in Google Scholar and ProQuest to capture grey literature, and the reference lists of the included studies were hand-searched. Detailed search strategies for each database are provided in S1 File.

### Study selection

The study selection process was conducted systematically to minimize bias and to ensure reproducibility. All records identified through the database searches were imported into Rayyan software, and duplicate entries were removed. In the ensuing screening phase, two independent reviewers (RA and KH) assessed the titles and abstracts of all unique records against the predefined eligibility criteria. Studies that appeared to meet these criteria or for which relevance was uncertain were advanced to a full-text review. In the subsequent stage, the same two reviewers independently examined the complete articles to determine the final inclusion. Any discrepancies arising at either the abstract or full-text screening levels were resolved through discussion until a consensus was reached or, when necessary, by arbitration from a third reviewer (EG).

### Data extraction

Data were systematically extracted using a customized data extraction form developed in Microsoft Excel. Two distinct forms were utilized: one for the primary outcome and the other for the secondary outcomes. The first form was designed to capture details including the author’s last name, publication year, study setting, design, sample size, quality score, and the prevalence of IPP-LARC, IPP-I, IPP-IUCD, EPP-LARC, EPP-I, and EPP-IUCD. The second form, for secondary outcomes, recorded the author’s last name, publication year, and data for the exposed and non-exposed- groups (including event rates and control groups).

Because outcome definitions varied across studies, we applied harmonization rules to ensure consistency in the definitions. Specifically, for studies conducted in the extended postpartum period, we extracted data on LARC methods (implants and IUCDs), as most of these studies reported the overall modern contraceptive utilization. For studies reporting LARC utilization without disaggregation by method, we extracted both IUCD and implant data where available. In addition, for some studies primarily assessing IUCD uptake, we also extracted information on implant use when reported to allow inclusion in pooled LARC estimates [51]. To ensure accuracy, the extraction process was also performed in duplicate by RA and KH.

### Quality appraisal

The methodological rigor of the included studies was evaluated using the Joanna Briggs Institute (JBI) critical appraisal checklist. This process was conducted independently by two reviewers (RA and KH), who assessed each primary study using the established criteria. A consensus was reached regarding the inclusion or exclusion of each article. In instances where the reviewers’ initial assessments diverged, the discrepancy was resolved by calculating and adopting the mean of their two individual scores. A study was classified as having a “low risk” of bias if it met more than 50% of the quality indicators on the relevant checklist.

The appraisal criteria were tailored to specific study designs. For cross-sectional- studies, quality was judged across eight domains, including the clarity of inclusion criteria, description of subjects and setting, validity of exposure measurement, objective outcome criteria, identification and management of confounders, and the appropriateness of statistical analysis. Case-control- studies were appraised against ten items, evaluating factors such as the comparability of groups, appropriateness of case and control selection, standardization of exposure measurement, handling of confounding variables, and the suitability of the statistical approach. Quasi-experimental studies (including pre–post intervention designs) were evaluated against nine items, covering clarity of cause-effect- relationships, similarity of participants, comparability of treatments, presence of control groups, multiple outcome measurements, completeness of follow-up-, consistency and reliability of outcome measurement, and appropriateness of statistical analysis.

Following this appraisal, 68 studies (63 cross-sectional-, three case–control, and two continuous quality improvement/quasi-experimental- studies) were evaluated. All studies achieved a quality score of 50% or higher, denoting a low risk of bias, and were consequently included in the subsequent analyses (S2 File).

### Data synthesis and analysis

A descriptive summary of the included studies was performed, detailing their characteristics, study populations, and reported outcomes. Stata version 17.0 (StataCorp LLC, College Station, TX, USA) and R version 4.5 (R Foundation for Statistical Computing, Vienna, Austria) were used for statistical analyses. The primary outcome of interest was the pooled prevalence of LARC use among postpartum women. Separate pooled prevalence estimates were computed for each outcome to allow comparisons across time frames and contraceptive methods. High heterogeneity was present in all analyses/pooled prevalence analyses. To address this between-study- variability, a random-effects- model was computed. The Freeman–Tukey double arcsine transformation was applied to stabilize the variance in prevalence estimates. Heterogeneity was quantified using the I² statistic, with values above 50% indicating substantial heterogeneity [52]. In this review, the I² values frequently exceeded 90%, underscoring the considerable methodological and contextual differences among the included studies. This persistently high heterogeneity is an important limitation and is explicitly acknowledged in the interpretation of the pooled estimates.

Subgroup analyses and meta-regressions- were conducted to explore potential sources of heterogeneity stratified by geographic region and study setting. Publication bias was assessed by visual inspection of funnel plots and Egger’s regression test [53]. Sensitivity analyses were performed to assess the influence of individual studies on pooled estimates, ensuring the robustness of the findings.

Temporal trends in postpartum contraceptive uptake were assessed using the non-parametric Mann–Kendall test. This test was applied to year-specific- pooled estimates for each outcome to detect monotonic increases or decreases over time. Tau (τ) statistics was reported, with p < 0.05 considered statistically significant. Linear regression plots were generated to visually illustrate the direction and magnitude of uptake trends across the study years.

### Certainty assessment

We assessed the certainty (confidence) of the body of evidence for each outcome using the Grading of Recommendations, Assessment, Development, and Evaluation (GRADE) approach. Certainty was based on five domains: risk of bias, evaluated using the JBI checklist; inconsistency, assessed by heterogeneity using the I² statistic; indirectness, which considered the applicability of the population, intervention, and outcomes; imprecision, based on the width of confidence intervals and sample size; and publication bias, examined using funnel plots and the Egger’s test.

### Results

A total of 1,085 records were identified: 415 from Hinari, 235 from PubMed, 98 from the Cochrane Library, 335 from Google Scholar, and 2 through manual searching. After removing 189 duplicates, 896 unique records were screened by title and abstract. Of these, 785 were excluded for not meeting inclusion criteria, primarily due to irrelevant populations, ineligible study designs, or outcomes unrelated to LARC. This left 111 full-text articles for eligibility assessment. Following full-text review, 41 further articles were excluded (29 for irrelevant outcomes, 2 for incorrect population, 2 for lack of full text, 2 for inappropriate design, 1 duplicate, and 7 for being outside the study scope). Ultimately, 68 studies met the inclusion criteria and were included in the final meta-analysis. S1 Fig presents a PRISMA flowchart summarising this process.

### Description of included studies

We identified 68 studies on immediate and extended PPFP across seven African countries. Together, these studies included a sample of 32,756 women, of whom 7,586 reported LARC use across both the immediate and extended postpartum periods. Most studies were conducted in Ethiopia (n = 53), with additional contributions from Kenya (n = 2), Uganda (n = 5), Rwanda (n = 2), Nigeria (n = 2), Burkina Faso (n = 1), and Ghana(n = 3). Most of the included studies employed cross-sectional designs, while a smaller number used case–control, pre-post, CQI, or mixed-method approaches. Sample sizes ranged from 105 [70] to 908 [67] participants, reflecting both facility- and community-based populations. Overall, 57.3% of the included studies focused on immediate (36 cross-sectional studies and three case-control studies) PPFP: 13 assessed LARC, 10 examined PPFP in general, 14 evaluated IUD uptake, and 2 focused on implant use. Among the 36 cross-sectional studies identified, 23 reported the prevalence of immediate postpartum LARC use, with rates ranging from 1.6% in Nigeria [63] to 53.2% in Ethiopia [55]. Implants were consistently the most frequently adopted method, with uptake as high as 50% of users in Kenya [73], while IPP-IUCD use varied widely, from less than 0.7% in Uganda to 21.9% in Ethiopia [78].

Twenty-nine studies were conducted during the extended postpartum period, including Eight on LARC, 18 on modern PPFP in general, one on implants, and one on IUD use. The prevalence of EPP-LARC estimates ranges between 1.5% [98] and 69.8% [93]. Similar to immediate uptake, implants dominated the method choice (Table 1).

**Table 1:** Description of included studies on Immediate/EPPLARC Studies in Eastern and Western Africa.

Author(s)	Year	Country	Region	SD	SS		ES [95% CI]			Quality score
						OV	LARC	Implant	IUCD	
1. Teshome et al. [54]	2025	Ethiopia	EA	CSS	317	IPP-LARC	35.3 [30.3-40.7]	30.3[25.5-35.5]	5.0 [3.1-8.0]	7
2. Arero et al. [55]	2022	Ethiopia	EA	CSS	393	IPP-LARC	53.2 [48.25-8.1]	47.6 [42.7,52.5]	5.8[3.9-8.6]	7
3. Belayihun et al. [21]	2021	Ethiopia	EA	CSS	884	IPP-LARC	36.3 [33-39.5]	11.3 [9.4-13.6]	1.8[1.1-2.9]	7.5
4. Gebremichael [56]	2019	Ethiopia	EA	CSS	693	IPP-LARC	17.5 [14.8-20.5]	–	–	8
5. Sori et al. [57]	2023	Ethiopia	EA	CQI	789	IPP-LARC	7.0 [5.4-8.9]	5.4[4.1-7.3]	1.5 [0.9-2.6]	8
6. Ayena et al. [58]	2023	Ethiopia	EA	CSS	411	IPP-LARC	27.3 [23.2-31.7]	22.4 [18.6-26.7]	4.9 [3.2-7.4]	8
7. Tariku et al. [59]	2022	Ethiopia	EA	CSS	418	IPP-LARC	25.4 [21.4-29.7]	19.4 [15.9-23.4]	6.0 [4.1-8.7]	7
8. Tegene et al. [60]	2025	Ethiopia	EA	CSS	421	IPP-LARC	43.9 [39.3-48.7]	42.0 [37.4-46.8]	1.9 [1.0-3.8]	7.5
9. Usso et al. [61]	2021	Ethiopia	EA	CSS	530	IPP-LARC	18.5 [15.5-22.0]	12.8 [10.2-15.9]	5.7 [4.0-7.8]	7
10. Bizuneh [62]	2022	Ethiopia	EA	CBC	341	IPP-LARC	33.7 [28.6-34.8]	30.8 [26.1- 57.4]	2.9 [1.6-5.3)]	8
11. Wudineh et al. [26]	2023	Ethiopia	EA	CSS	417	IPP-LARC	30.7 [26.5-35.3]	23.3 [19.5--27.5]	7.4 [5.3-10.4]	7
12. Kachiro et al. [63]	2021	Nigeria	WA	CSS	123	IPP-LARC	1.6 [0.4-5.7]	–	–	7.25
13. Demissie et al. [64]	2019	Ethiopia	EA	CSS	586	IPPFP	13.0[10.5-15.9]	6.14 [4.5-8.4]	3.7 [2.5-5.6]	7.5
14. Gudeta et al. [23]	2025	Ethiopia	EA	CBC	836	IPPFP	15.5[13.2-18.2]	15.5 [13.2-18.2]	2.8 [1.8-4.1]	7.75
15. Asnake et al. [65]	2021	Ethiopia	EA	CBC	884	IPPFP	44.1[40.9- 47.4]	32.9 [29.9-36.1]	--	8
16. Silesh et al. [66]	2022	Ethiopia	EA	CSS	394	IPPFP	21.3[17.6- 25.6]	17.3 [13.8-21.3]	2.8 [1.6-4.9]	8
17. Sium et al. [67]	2022	Ethiopia	EA	QES	908	IPPFP	15.4[13.2-17.9]	13.3 [11.3-15.7]	2.0 [1.3-3.1]	8
18. Tesfaye et al. [68]	2023	Ethiopia	EA	CSS	226	IPPFP	36.7[30.7- 43.2]	24.3 [19.3-30.3]	12.4 [8.7-17.2]	7.5
19. Gadigbe et al. [69]	2025	Burkina Faso	WA	CSS	853	IPPFP	31.6 [28.6-34.8]	21.9 [19.3-24.8]	9.7 [7.9- 11.9]	7.25
20. Kitessa et al. [70]	2019	Rwanda	EA	CSS	105	IPPFP	7.6 [3.6,14.3]	–	–	7.5
21. Nakiwunga et al. [71]	2022	Uganda	EA	CSS	397	IPPFP	12.6 [97-16.2]	12.6 [9.7-16.2]	–	7.25
22. Obua et al. [72]	2022	Uganda	EA	CSS	300	IPPFP	2.0 [0.9-4.3]	1.3 [0.5-3.4]	0.7 [0.2-2.4]	7.25
23. Shabiby et al. [73]	2015	Kenya	EA	CSS	185	IPPI	–	50.3 [43.1-57.4]	–	7.25
24. Mogeni et al. [74]	2019	Kenya	EA	CSS	353	IPPI	–	44.2 [39.1-49.4]	–	8
25. Melkie et al. [51]	2021	Ethiopia	EA	CSS	423	IPP-IUCD	9.7 [7.23-12.9]	5.7 [3.8-8.3]	4.0 [2.5, 6.3]	7.5
26. Abdullahi et al. [75]	2024	Ethiopia	EA	CSS	412	IPP-IUCD	–	–	10.9 [8.3, 14.3]	7.5
27. Aemro et al. [40]	2022	Ethiopia	EA	CSS	488	IPP-IUCD	–	–	22.1[18.7,16.0]	7.5
28. Dinsa et al. [76]	2024	Ethiopia	EA	CSS	290	IPP-IUCD	–	–	19.3[15.2, 24.2]	7.5
29. Geda et al. [22]	2021	Ethiopia	EA	CSS	286	IPP-IUCD	–	–	14.8[11.0, 19.3]	7.5
30. Guye et al. [42]	2023	Ethiopia	EA	CSS	605	IPP-IUCD	–	–	27.3[23.9, 31.0]	7.5
31. Hagos et al. [77]	2020	Ethiopia	EA	CSS	182	IPP-IUCD	–	–	3.3[1.5, 7.0]	6.25
32. Tefera et al. [78]	2017	Ethiopia	EA	CSS	310	IPP-IUCD	–	–	21.9[17.7, 26.9]	7.5
33. Gebremedhin et al. [79]	2019	Ethiopia	EA	CSS	452	IPP-IUCD	–	–	13.7[10.8, 17.2]	7.5
34. Kanakuze et al. [80]	2020	Rwanda	EA	MM	383	IPP-IUCD	–	–	28.2[23.9, 32.9]	7.25
35. Shiferaw et al. [81]	2023	Ethiopia	EA	CSS	392	IPP-IUCD	–	–	9.5[7.4, 13.3]	8
36. Alupo et al. [82]	2024	Uganda	EA	MM	422	IPP-IUCD	–	–	2.4 [1.3, 4.3]	7.5
37. Eristu et al. [49]	2024	Ethiopia	EA	CBCS	617	EPP-LARC	36.3 [32.6-40.2]	31.1 [27.6-34.9]	5.2 [3.7-7.2]	8
38. Mesfin et al. [24]	2021	Ethiopia	EA	CBCS	416	EPP-LARC	22.6 [18.8-26.9]	18.5 [15.1-22.5]	4.1 [2.3-6.4]	7
39. Tamrie et al. [83]	2015	Ethiopia	EA	CBCS	441	EPP-LARC	36.7 [32.3- 41.3]	42.2 [37.6-46.8]	3.2 [1.9-5.3]	8
40. Woldu et al. [25]	2020	Ethiopia	EA	CSS	381	EPP-LARC	36.5 [31.8-41.4]	34.1 [29.5-39.0]	2.4 [1.2-4.4]	7.5
41. Aliyi [84]	2017	Ethiopia	EA	CBCS	783	EPP-LARC	7.3 [5.7-11.0]	5.9 [4.4-7.7]	1.4 [0.8-2.5]	8
42. Appiah et al. [85]	2024	Ghana	WA	CSS	406	EPP-LARC	3.9 [2.4- 6.3]	–	–	7.25
43. Negash [50]	2020	Ethiopia	EA	CBCS	602	EPP-LARC	33.2 [29.6-37.1]	30.9 [27.3-34.7]	2.3 [1.4-3.9)	7.25
44. Anguzu et al. [86]	2018	Uganda	EA	CBCS	400	EPP-LARC	8.5[6.1-11.6]	6.7 [4.9-9.6]	1.7 [0.8-3.6]	8
45. Abraha et al. [87]	2017	Ethiopia	EA	CBCS	590	EPPFP	–	11.9 [9.5-14.7]	–	7.25
46. Assefa et al. [88]	2021	Ethiopia	EA	CBCS	402	EPPFP	27.9 [23.7-32.4]	21.1 [17.4-25.4]	6.7 [4.7-9.6]	8
47. Abraha et al. [89]	2018	Ethiopia	EA	CBCS	905	EPPFP	18.2 [15.8 - 20.9]	13.8 [11.7-16.2]	4.4 [3.3-5.7]	8
48. Gejo et al. [90]	2019	Ethiopia	EA	CBCS	368	EPPFP	31.2 [26.7-36.1]	24.5 [20.3-29.1]	6.8 [4.6-9.8]	7.5
49. Asah-Opuka et al. [91]	2019	Ghana	WA	RCSS	565	EPPFP	–	69.2 [65.3-72.9]	–	7
50. Abebe et al. [92]	2023	Ethiopia	EA	CBCS	385	EPPFP	13.5 [10.4-17.3]	11.7 [8.8-15.3]	1.8 [0.9-3.7]	7.25
51. Wekere et al. [93]	2024	Nijeria	WA	CBCS	609	EPPFP	69.8 [66.0-73.3]	48.6 [44.7-52.6]	21.2 [18.1-24.6]	7.5
52. Agula et al. [94]	2022	Ghana	WA	CBCS	624	EPPFP	22.6(19.5-26.0]	17.1 [29.4-39.0]	5.4 [3.9-7.5]	7.25
53. Jaleta et al. [95]	2024	Ethiopia	EA	CSS	422	EPPFP	16.6 [13.4- 20.4]	13.5 [10.6-17.1]	3.1 [.8-5.2]	8
54. Getaneh et al. [96]	2021	Ethiopia	EA	CBCS	619	EPPFP	5.8 [4.2- 7.9]	5.8 [4.2-7.9]	–	7.5
55. Kenate & Amenu [97]	2015	Ethiopia	EA	CBCS	120	EPPFP	10.8 [6.4-17.7]	6.7 [3.4-12.6]	4.2 [1.8-9.4]	7.25
56. Niguse et al. [98]	2019	Ethiopia	EA	CBCS	505	EPPFP	1.98 [1.02- 17.7]	1.6 [0.8-3.1]	0.4 [0.1-1.4]	8
57. Nugussa et al. [99]	2023	Ethiopia	EA	CBCS	385	EPPFP	34.0 [29.5- 38.9]	21.3 [17.5-25.7]	12.7 [9.8-16.4]	8
58. Tafa & Worku [100]	2018	Ethiopia	EA	CBCS	625	EPPFP	32.5 [28.9- 36.2]	20.6 [17.6-24.0]	11.8 [9.5-14.6]	8
59. Gebremedhin et al. [101]	2018	Ethiopia	EA	CBCS	803	EPPFP	27.5 [24.5- 30.7]	7.5 [5.8-9.5]	2.0 [1.2-3.2]	7.25
60. Mengesha et al. [102]	2015	Ethiopia	EA	CBCS	899	EPPFP	2.0 [1.3-3.14]	0.2[0.06-0.8]	1.8 [1.1-2.9]	8
61. Andualem et al. [103]	2022	Ethiopia	EA	CBCS	400	EPPFP	23.5 [19.6-27.9]	19.2[15.7-23.4]	4.2 [2.7-6.7]	8
62. Mihretie et al. [104]	2020	Ethiopia	EA	CBCS	402	EPPFP	8.0 [5.7-11.0]	6.0 [4.0-8.7]	2.0 [1.0-3.9]	7.25
63. Ashebir et al. [105]	2020	Ethiopia	EA	CBCS	681	EPPFP	–	5.9 [4.3-7.9]	–	8
64. Nigussie et al. [106]	2016	Ethiopia	EA	CBCS	545	EPPFP	7.7 [5.7-10.2]	4.8 [3.3-6.9]	2.9 [1.8-4.7]	8
65. Omona & Namuli [107]	2020	Uganda	EA	MM	202	EPP-IUCD	–	–	12.7 [9.8-16.4]	8
**Case studies**										
66. Adella et al. [108]	2024	Ethiopia	EA	CCS	324	IPP-LARC	–	–	–	9
67. Assefaw et al. [109]	2021	Ethiopia	EA	CCS	420	IPP-IUCD	–	–	–	8.75
68. Seid et al. [110]	2020	Ethiopia	EA	CCS	510		–	–	–	9

CSS: Facility-based cross-sectional studies; CBCS: Community cross-sectional studies; QES: quasi-experimental study; CQI: continuous quality intervention; SS: sample size; SD: study design; OV: outcome variable; EPPFP: Extended postpartum FP; IPPFP: immediate postpartum FP.

### Prevalence of IPP-LARC uptake

Among the 36 cross-sectional studies indentified during the immediate postpartum period, 23 defined IPP-LARC uptake as the adoption of either an implant or an IUCD, yielding a pooled prevalence of 21.9% (95% CI:16.4–28.4). Method-specific uptake was further stratified based on available disaggregated data: among the 36 identified studies, only 22 separately reported the prevalence of IPP-implant (IPP-I) use, with a pooled prevalence of 20.5% (95% CI:14.8–27.7), while 29 studies reported on IPP-IUD use, yielding a pooled prevalence of 7.4% (95% CI:5.1–10.6). It should be noted that these method-specific figures are derived from different subsets of the included literature; consequently, the pooled prevalence for the overall LARC category is not a simple additive total of the individual implant and IUCD estimates (S2–S4 Figs).

### Prevalence of EPP-LARC uptake

The pooled prevalence of EPP-LARC uptake in Eastern and Western Africa was estimated using 25 studies (S5 Fig). The pooled prevalence of EPP-LARC uptake was 19.5%, with individual study estimates ranging from 1.98% [98] to 69.8% [93] across all countries. Method-specific uptake was further stratified: 27 studies contributed data on EPP-I uptake, with a pooled prevalence of 16.2% (S7 Fig), and study-specific estimates ranged from 0.2% [102] to 69.2% [91]. Similarly, 24 studies reported on EPP-IUD use—among other modern contraceptive options—yielding a pooled prevalence of 4.5% for this specific method (S7 Fig), with a prevalence ranging from 0.4% to 21.2% across the included studies [93,98].

### Subgroup analysis

Overall, IPP-LARC uptake was slightly higher in Western Africa (26.3%) than in Eastern Africa (22.6%), with Ethiopia reporting consistently higher levels within Eastern Africa, while Uganda and Rwanda showed lower uptake. In Western Africa, Burkina Faso demonstrated a relatively high prevalence, whereas Nigeria reported the lowest. For EPP-LARC, uptake was again higher in Western Africa (28.4%) than in Eastern Africa (18.3%), with Ethiopia showing moderate levels, Uganda showing lower uptake, Nigeria showing the highest uptake, and Ghana reporting a more modest prevalence (13.7%). Further details are provided in Table 2.

**Table 2:** Pooled estimates for postpartum contraceptive uptake by region/country.

Region/ Country	ES (95% CI)/I^2^(%)					
	IPP-LARC	EPP-LARC	IPP-I	EPP-I	IPP-IUCD	EPP-IUCD
Eastern Africa	22.6 [16.9–28.9]/ 98.2	18.3 [12.9–24.4] / 98.5	20.4 [14.8–26.6]/ 98.2	13.4 [9.13–18.4] / 98.3	7.3 [4.8–10.2]/ 97.1	3.9 [2.7–5.4]/ 91.8
Western Africa	26.3 [23.6–29.2]	28.4 (11.4–71.1]	21.9 [19.2–24.8]	44.2 [15.9–74.7]	9.7 [7.9–11.9]	12.1 [10.3-14.0]
Ethiopia	25.8 (19.7, 32.5) /98.2	18.9 (13.2–22.0) / 98.5	19.8 [14.4–26.0]/ 98.1	13.7 [9.2–18.9]/ 98.4	7.3 [4.9–10.2]/ 98.1	3.7 [2.5–5.0]/ 91.0
Uganda	7.03 [5.2–9.0]	8.5 [6.2–11.6]	6.5 [4.8–8.5]	6.7 [4.7–20.3]	1.5 [0.7–2.6]	5.1 [3.5–7.0]
Rwanda	7.6 [3.9–14.3]	–	–	28.2 [23.9–32.9]	–	–
Burkina Faso	31.6 [28.7–34.8]	–	21.9 [19.3–24.8]	–	9.3 [7.9–11.9]	–
Nigeria	1.6 [0.4–5.7]	69.8 [66.0–73.3)	–	48.6(44.6–52.54]	–	21.2[18.1–24.6]
Ghana	–	13.7 [11.6–16.8]	–	40.6 [37.8–43.4	–	5.4(3.9–7.5]
Kenya	–	–	4.5 [2.8–8.4]	–	–	–

### Meta-regression

Meta-regression- analyses incorporating study year and sample size did not identify significant moderators of heterogeneity across outcomes, except for EPP-IUCD, where the year of publication was a source of heterogeneity. The coefficients for both covariates were consistently non-significant-, indicating substantial unexplained between-study- variance. This suggests that neither temporal trends nor study size accounted for the observed heterogeneity, reinforcing the need for further exploration of contextual or methodological factors that may explain the differences in postpartum LARC uptake across studies (Table 3).

**Table 3:** Meta-regression analysis of factors affecting between-study heterogeneity.

Outcome	Covariate	Coefficient	St. error	p > |z|	[95% CI]
IPPL-ARC	Sample	0.0057	0.0120	0.63	[-0.018–0.0292]
	Year	2.8043	1.6549	0.09	[-0.439–6.048]
EPP-LARC	Sample	4.36e-07	0.0002	1.00	[-0.0004–0.0004]
	Year	0.0058	0.0124	0.64	[-0.0184– 0.0301]
IPP-I-	Sample	−0.0199	0.0133	0.14	[-0.046–0.0062]
	Year	−0.6857	1.3720	0.61	[-3.374–2.003]
EPP-I-	Sample	−0.0001	0.0002	0.67	[-0.0004–0.0003]
	Year	0.0132	0.0117	0.26	[-0.0098–0.0363]
IPP-IUCD	Sample	−0.0074	0.0074	0.31	[.0219–0.0070]
	Year	−0.8238	0.7903	0.30	[-2.373-0.725]
EPP-IUCD	Sample	1.87e-06	0.0001	0.97	[-0.0001–0.0001]
	Year	0.0076	0.003791	0.04	[0.0001–0.0150]

### Publication bias

Visual inspection of funnel plots for all outcomes suggested approximate symmetry, with no clear evidence of distortion (S8 Fig). Consistent with this, Egger’s regression test was non-significant- across all analyses (IPP-LARC: p = 0.571; EPP-LARC: p = 0.71; IPP-I: p = 0.143; EPP-I: p = 0.31; IPP-IUCD: p = 0.123; EPP-IUCD: p = 0.40), indicating no statistical evidence of small study- effects or publication bias. Begg’s test also did not detect significant asymmetry. Taken together, these findings suggest that both visual inspection and regression-based- diagnostics support the absence of publication bias in the included studies.

### Sensitivity analysis

Sensitivity analyses were performed for all outcome variables (IPP-LARC, IPP-I, IPP-IUCD, EPP-LARC, EPP-I, and EPP-IUCD) to examine the impact of each individual study on the overall meta-analysis summary estimate. The omission of individual studies did not substantially alter the pooled estimates, indicating that the overall results were robust and not unduly influenced by any single study (S1–S6 Tables)

### Trend analysis of postpartum contraceptive uptake

The Mann–Kendall test demonstrated a significant positive trend in IPP-LARC uptake over time (τ = 1, p = 0.027), indicating consistent increases across study years. EPP-LARC showed a weak positive trend (τ = 0.289, p = 0.283), which was not statistically significant. IPP-I exhibited no evidence of a monotonic trend (τ = 0.067, p = 1), suggesting stability across study years. For EPP-I, the test revealed a moderate positive trend (τ = 0.422, p = 0.107), which did not reach statistical significance. IPP-IUCD uptake showed no significant monotonic change (τ = –0.357, p = 0.266), reflecting variability without a clear directional pattern. By contrast, EPP-IUCD demonstrated a significant positive trend (τ = 0.644, p = 0.012), indicating consistent increases across study years (S9 Fig).

#### Determinants of IPP-LARC, IPP-IUCD and EPP-LARC uptake in Eastern and Western Africa.

As shown in Table 4, several determinants were significantly associated with postpartum contraceptive uptake. Counseling consistently emerged as one of the strongest predictors across all outcomes. Women who received FP counseling were over three times more likely to adopt IPP-LARC (OR=3.5; 95% CI:2.0–6.17), more than four times more likely to adopt IPP-IUCD (OR=4.7; 95% CI:2.3–9.3), and more than three times more likely to adopt EPP-LARC (OR=3.2; 95% CI:2.7–3.9). Partner involvement demonstrated the largest effect size for IPP-LARC uptake (OR=5.6; 95% CI:3.0–10.4). Good knowledge was another consistent determinant, increasing the likelihood of uptake for both IPP-LARC (OR=3.5; 95% CI:1.8–6.9) and EPP-LARC (OR=3.7; 95% CI:2.9–4.8). Previous family planning history was associated with higher adoption of EPP-LARC (OR=3.1; 95% CI:1.7–5.8). Women who did not desire more children were 2.4 times more likely to adopt IPP-LARC compared to those who did (OR=2.4; 95% CI:1.8–2.9). Similarly, higher maternal education was positively associated with IPP-IUCD use (OR=1.7; 95% CI:1.2–2.4). Furthermore, mothers aged ≥35 years were 2.2 times more likely to adopt an IPP-IUCD specifically, compared to those aged ≤24 years (OR=2.2; 95% CI:1.3–3.7).

**Table 4:** Determinants of IPP-LARC, EPP-LARC and IPP-IUCD uptake in Eastern and western Africa.

Outcome & Determinant	Comparison	NS*	OR (95% CI)	I^2^ (%)	Egger’s test (p-value)
**IPPLARC uptake**					
Counselling abut FP during MCH [21,23,26,54,55,58–62,66,108]	Yes vs No	12	3.5 [2.1–65.7] *	89.9	0.779
Partner involvement [26,54,58,60,61,66]	Yes vs No	6	5.6 [31–10.3] *	85.9	N/A
Previous FP history [21,26,54,55,59,61,66,78]	Yes vs No	8	1.7 [0.95–2.97]	90.4	N/A
Knowledge [23,58,61,66,108]	Good vs poor	5	3.5 [1.6–7.7] *	88.5	N/A
Attitude [23,26,58,66,108]	Positive vs negative	5	1.6 [0.5–4.8]	95.9	N/A
Desire to fertility [54,55,60,66,108]	No vs Yes	5	2.3 [1.5–3.8] *	68.8	N/A
Mode of delivery [21,54,55,61,62,66]	CD vs NSVD	6	1.8 [0.8–4.0]	90.7	N/A
Age [26,55,59,62,66]	≥ 35 vs ≤ 24 years	5	1.4 [0.7–3.0]	78.1	N/A
Age [26,55,59,62,66]	25-34 vs ≤ 24 years	5	1.2 [0.9–1.6]	36.6	N/A
**IPP-IUCD uptake**					
Counselling abut FP during MCH [22,40,42,51,62,64,76,109,110]	Yes vs No	9	4.7 [2.2–10.0] *	90.1	N/A
Knowledge [22,40,42,64,78,109]	Good vs poor	6	3.5[1.9–6.9]]	88.8	N/A
Attitude [40,42,64,109]	Positive vs negative	4	1.5 [0.6–3.4]	94.4	N/A
Partner involvement [40,42,64,81,110,111]	Yes vs No	6	2.0 [0.8–5.4]	95.9	N/A
Age [22,42,51,64,75,76,109]	≥ 35 vs ≤ 24 years	7	2.2 [1.3–3.7] *	50.3	N/A
Age [22,42,51,64,75,76,109]	25-34 vs ≤ 24 years	7	1.5 [0.9–3.6]	71.5	N/A
Education [22,42,64,76,109]	Higher vs no education	5	1.7 [1.2–2.4] *	10.1	N/A
Education [22,42,64,76,109]	Secondary vs no education	5	2.0 [0.9–4.3]	82.7	N/A
Education) [22,42,64,76,109]	Primary vs no education	5	1.2 [0.9–1.7]	0	N/A
**EPPL-ARC uptake**					
Counselling [24,25,50,83,84]	Yes vs No	5	3.2 [25–4.2] *	41.5	N/A
Previous use [25,50,83,84]	Yes vs No	4	3.1 [1.67–6.0] *	85.5	N/A
Knowledge [24,25,49]	Good vs poor	3	3.7 [2.9–4.7] *	0	N/A

* Significant at P-value< 0.05, N/A: not applicable, Number of studies (n = 9) too small to test for small study effects (n.min = 10).

In contrast to these significant predictors, other factors such as maternal attitude and mode of delivery did not show a statistically significant association with uptake. Specifically, no significant impact was found for attitudes toward IPP-LARC (OR=1.6; 95% CI:0.5–4.8) or IPP-IUCD (OR=1.5; 95% CI:0.6–3.4), nor for the mode of delivery regarding IPP-LARC (OR=1.8; 95% CI:0.8–4.0). Furthermore, maternal age was not a significant determinant for IPP-LARC across any category, including the ≥ 35 group (OR=1.4; 95% CI:0.7–3.0) and the 25–34 group (OR=1.2; 95% CI:0.9–1.6). Similarly, the 25–34 age group showed no significant association with IPP-IUCD use (OR=1.5; 95% CI:0.9–3.6), indicating that the influence of age was limited strictly to the ≥ 35 cohort for that specific method.

### Certainty of evidence

Certainty of evidence for each outcome was assessed using the GRADE approach. All pooled prevalence outcomes (IPPLARC, IPP-IUCD, EPPLARC) were rated very low certainty due to serious inconsistency (high heterogeneity), despite no serious concerns for risk of bias, indirectness, imprecision, or publication bias. Determinants such as counseling and partner involvement showed low to moderate certainty, depending on heterogeneity.

## Discussion

This systematic review and meta-analysis synthesize evidence on immediate and extended postpartum uptake of LARCs across Eastern and Western Africa. The pooled prevalence estimates—21.8% for immediate postpartum use and 19.5% for extended postpartum use—indicate that while postpartum contraception is increasingly adopted, overall uptake remains below global recommendations. Stratification of outcomes into immediate and extended postpartum periods highlights important differences: motivation and opportunity are highest immediately after delivery, yet continuity of use during the extended postpartum period is weaker, reflecting gaps in follow-up care and service integration. Trend analysis further revealed significant increases in IPP-LARC and EPP-IUCD uptake across study years, while other outcomes (EPP-LARC, IPP-I, EPP-I, IPP-IUCD) showed no statistically significant monotonic change, underscoring persistent barriers to sustained growth.

Geographic variation was particularly notable. Most studies originated from Eastern Africa, especially Ethiopia, where uptake rates were relatively higher than in other regions. IPP-LARC prevalence in Eastern Africa was 22.6%, compared to 26.3% in Western Africa. Country-level differences were striking: Ethiopia reported 25.8%, while Uganda and Rwanda showed much lower uptake (7.0% and 7.6%, respectively). In contrast, Burkina Faso demonstrated relatively high uptake (31.6%), whereas Nigeria reported extremely low levels (1.6%). EPP-LARC uptake followed a similar pattern, with Eastern Africa at 18.3% compared to 28.4% in Western Africa. Nijeria showed higher uptake (69.8%), while Uganda reported only 8.5%. These regional disparities, combined with the temporal trends, suggest that while certain methods are gaining traction, others remain stagnant, reflecting uneven progress across countries and contraceptive types. These variations are likely influenced by underlying health system factors, such as the extent to which postpartum LARC services are integrated into public insurance schemes or offered at no cost to the user. High upfront costs remain a significant barrier in many settings, and the inconsistent availability of trained providers and necessary commodities across all maternity facilities—rather than just tertiary centres—further restricts equitable access.The lack of significant upward trends in IPP-IUCD and EPP-I uptake reinforces this underutilization, highlighting the need for targeted interventions to normalize IUCD provision in postpartum care and and to align policy environments with clinical needs.

Method-specific differences further supplemented the findings. Implants were consistently more utilized than IUCDs across both immediate and extended postpartum periods. Immediate postpartum implant use reached 20.4% in Eastern Africa and 21.9% in Western Africa, compared to only 7.3% and 9.7% for IPP-IUCDs. Extended postpartum implant use was also higher (13.7% in Eastern Africa; 44.2% in Western Africa) than EPP-IUCD use (3.9% and 12.1%, respectively). This preference may be explained by provider familiarity and perceived ease of insertion. Furthermore, lower uptake of IUCDs is often driven by a greater fear of side effects and deep-seated cultural misconceptions compared to implants. Despite their proven efficacy and cost-effectiveness, IUCDs remain underutilized, representing a missed opportunity for expanding postpartum contraceptive options.

Taken together, these disparities suggest that health system capacity and policy environments strongly shape postpartum contraceptive adoption. Addressing provider diffidence and logistical barriers through targeted training and improved service integration could help rebalance method choice and expand the range of options available to women. Strengthening follow-up care is also critical to sustaining uptake during the extended postpartum period. The observed significant upward trends for IPP-LARC and EPP-IUCD demonstrate that progress is possible when health systems prioritise integration and counselling, offering a pathway for scaling other methods across the region.

This review revealed that counseling provided during maternal and child health services was one of the strongest predictors of IPP-LARC, IPP-IUCD, and EPP-LARC uptake, consistent with findings from systematic reviews and meta-analyses conducted in SSA and LMICs [112,113]. Structured counseling plays a critical role in dispelling misconceptions, enhancing informed choice, and integrating contraceptive decision-making into routine maternal care [114]. Male partner involvement emerged as an even more influential factor, significantly increasing uptake of IPP-LARC, thereby underscoring the importance of shared decision-making and the need to address gender dynamics in family planning programs [115–117]. This conclusion aligns with evidence from systematic reviews and meta-analyses conducted in Ethiopia [66]. In addition, previous family planning use was identified as a significant determinant of EPP-LARC adoption, suggesting that prior exposure to contraceptive methods builds confidence and reduces barriers to postpartum uptake.

Maternal age was identified as another important determinant of IPP-IUCD uptake, a finding consistent with results from studies conducted in five LMICs [17]. A possible explanation is that, as women grow older, they are more likely to have reached their desired family size and thus demonstrate a greater need for effective long-term contraception. Additionally, older women may have accumulated more knowledge and experience regarding reproductive health throughout their lives compared to younger women. This maturity may result in a reduced fear of potential side effects and greater confidence in managing them, which could further contribute to the higher uptake rates of the IUCD observed in this group. Knowledge was also associated with uptake: women with better knowledge and more positive attitudes had significantly higher odds of adopting IPP-LARC and EPP-LARC. This pattern appears universal, as improved knowledge is linked to increased utilization of maternal and reproductive health services. These findings justify investments in community education and demand-generation strategies, as knowledge and attitudes are modifiable through targeted interventions, suggesting that sustained engagement and follow-up are essential to maintain uptake beyond the immediate postpartum period.

Fertility desire was another important determinant, with women expressing a desire to delay or limit fertility showing significantly higher uptake, reflecting the alignment of contraceptive use with reproductive intentions.

### Strengths

The manuscript benefits from a large pooled sample size, clear differentiation between immediate and extended postpartum periods, method-specific analyses, and rigorous sensitivity and publication bias assessments. The findings are highly relevant for policy and clinical practice in Africa.

### Limitations of the study

The dominance of studies from Ethiopia limits the generalisability of the findings to other African sub-regions. Furthermore, as most included studies utilised a cross-sectional design, causal inference is restricted. Despite performing subgroup and meta-regression analyses, high heterogeneity remains a significant concern. Regarding quality assessment, studies were classified as low risk of bias if they met ≥ 50% of the JBI appraisal criteria. While this pragmatic threshold ensured the inclusion of a broad evidence base, it may be considered lenient and should be interpreted as a methodological limitation. In addition, this review primarily focused on quantitative determinants of uptake; therefore, the influence of deep-seated cultural norms and individual beliefs was not formally analysed. As these factors are typically explored through qualitative methodologies, their impact on the observed geographic variations remains an area for further investigation

#### Clinical implications.

The findings carry important implications for clinical practice. Immediate postpartum insertion of LARCs should be normalized as part of routine obstetric care, particularly during facility-based deliveries where women are already in contact with the health system. Clinicians require enhanced training not only in insertion techniques but also in counseling skills, especially for IUDs, to overcome provider hesitancy and expand method choice. Patient-centered counseling is critical, with emphasis on the safety, reversibility, and convenience of LARCs, as well as addressing misconceptions and fears that often deter uptake. Structured follow-up visits within the first year postpartum can help reduce discontinuation rates, manage side effects, and reinforce satisfaction, thereby improving continuation and long-term effectiveness. By embedding postpartum LARC provision into routine maternal and newborn care, clinicians can play a pivotal role in reducing unintended pregnancies and improving maternal and child health outcomes.

#### Policy implications.

From a policy perspective, the evidence underscores the urgent need to strengthen health system integration of PPFP. Ministries of Health should embed LARC provision into maternal and newborn care packages, ensuring continuity from antenatal counseling through delivery and postnatal follow-up. Resource allocation must prioritize supply chain strengthening to prevent stock-outs of implants and IUDs, thereby ensuring equitable access across both urban and rural facilities. Policies should also encourage male partner involvement in postpartum contraceptive counseling, as evidence demonstrates that partner support improves uptake. National health information systems should disaggregate postpartum contraceptive data by timing and method, enabling more precise monitoring of progress toward WHO-recommended birth intervals. Finally, equity-focused strategies are essential to reach underserved populations, including rural women, adolescents, and those with limited education, thereby reducing disparities in access and utilization.

### Conclusion

This review demonstrates that postpartum LARC uptake in Eastern and Western Africa remains suboptimal, with significant regional and method-specific variations. Implants dominate utilization, while IUDs remain underused despite their proven efficacy. The findings highlight missed opportunities at facility-based deliveries and during the extended postpartum period, where unmet need for FP is highest. Strengthening integration of LARCs into maternal health services, enhancing provider capacity, and addressing socio-cultural barriers are essential to improving uptake. Policy action that prioritizes postpartum contraception will be pivotal in reducing unintended pregnancies, advancing maternal and child survival, and achieving reproductive autonomy across the continent. By consolidating fragmented evidence into a robust synthesis, this study provides a critical foundation for clinical practice and policy formulation aimed at transforming maternal health outcomes in study area.

## Supporting information

S1 File Search term in each data base. (DOCX)

S2 File Quality appraisal of included studies. (DOCX)

S1 Table Leave-One-Out Meta-Analysis for Pooled Prevalence of IPP-LARC. (DOCX)

S2 Table Leave-One-Out Meta-Analysis for Pooled Prevalence of IPP-I. (DOCX)

S3 Table Leave-One-Out Meta-Analysis for Pooled Prevalence of IPP-IUD. (DOCX)

S4 Table Leave-One-Out Meta-Analysis for Pooled Prevalence of EPP-LARC. (DOCX)

S5 Table Leave-One-Out Meta-Analysis for Pooled Prevalence of EPP-I. (DOCX)

S6 Table Leave-One-Out Meta-Analysis for Pooled Prevalence of EPPIUCD. (DOCX)

S1 Fig PRISMA flow diagram of study selection for immediate and extended postpartum LARC uptake in Eastern Africa and Western Africa, 2025. (DOCX)

S2 Fig Pooled prevalence of IPP-LARC uptake in Eastern and Western Africa, 2025. (DOCX)

S3 Fig Pooled prevalence of IPP-I uptake in Eastern and Western Africa, 2025. (DOCX)

S4 Fig Pooled prevalence of IPP-IUD uptake in Eastern and Western Africa, 2025. (DOCX)

S5 Fig Pooled prevalence of EPP-LARC uptake in Eastern and Western Africa, 2025. (DOCX)

S6 Fig Pooled prevalence of EPP-I uptake in Eastern and Western Africa, 2025. (DOCX)

S7 Fig Pooled prevalence of EPP-IUD uptake in Western and Eastern Africa, 2025. (DOCX)

S8 Fig Funnel plots assessing publication bias across immediate and early postpartum contraceptive outcomes: (a) IPP-LARC, (b) EPP-LARC, (c) IPP-I, (d) EPP-I, (e) IPP-IUD, (f) EPP -IUD. (DOCX)

S9 Fig Trends in postpartum contraceptive uptake across study years based on mean effect sizes: (a) IPP-LARC, (b) EPP-LARC, (c) IPP-I, (d) EPP-I, (e) IPP-IUCD, and (f) EPP-IUCD. Significant upward trends were observed for IPP-LARC and EPP-IUCD, while EPP-LARC, IPP-I, EPP-I, and IPP-IUCD showed no statistically significant monotonic change.
