# Supplementary material for: Uptake and determinants of immediate and extended postpartum long-acting reversible contraceptive use in Eastern and Western Africa: A systematic review and meta-analysis
Source: PLoS One. 2026 Apr 17;21(4):e0346885. doi: 10.1371/journal.pone.0346885 (PMC13089893; doi:10.1371/journal.pone.0346885)
Supplement: S1 File — (DOCX) [file pone.0346885.s001.docx]

**S1 File.** Search term in each data base.

| **Database** | **Search** | **Result** |
| --- | --- | --- |
| **PubMed** | (((((((((("Postpartum Period"[Mesh]) OR ("After childbirth"[Text Word])) OR ("Postpartum"[Text Word])) OR ("Extended postpartum period"[Text Word])) OR ("Postnatal"[Text Word])) OR ("Immediate postpartum"[Text Word]))) OR ("puerperium"[Text Word])) AND (((((((((((((((("Long-Acting Reversible Contraception"[Mesh]) OR ("long acting reversible contracepti*"[Text Word])) OR ("LARC"[Text Word])) OR ("Intrauterine device"[Text Word])) OR ("IUCD"[Text Word])) OR ("IUD"[Text Word])) OR ("Implant"[Text Word])) OR ("Contraceptive Implant"[Text Word])) OR ("Implanon"[Text Word])) OR ("jadelle"[Text Word])) OR ("Family planning"[Text Word])) OR ("family planning services"[Text Word])) OR ("postpartum Family Planning"[Text Word])) OR ("Contraception"[Text Word])) OR ("Contraception"[Text Word])) OR ("Family Planning Services"[Mesh]))) AND ((((((((("Uptake"[Text Word]) OR ("Use"[Text Word]))) OR ("Utilization"[Text Word])) OR ("Adoption"[Text Word])) OR ("Coverage"[Text Word])) OR ("Contraceptive use")) OR ("Service uptake"[Text Word])) OR ("Adoption"[Text Word]))) AND (((("East Africa"[Text Word]) OR ("West Africa"[Text Word])) OR ("Africa, Eastern"[Mesh])) OR ("Africa, Western"[Mesh])). Filter: Full text, from 2015/1/1 - 2025/10/12 | **235** |
| **Hinari** | ("immediate postpartum" OR postnatal OR postpartum OR “Extended postpartum”) AND ("long-acting reversible contraceptive" OR LARC OR implant OR IUD OR IUCD OR Implanon OR Jadelle OR "family planning services" OR "maternal health" OR "primary health care") AND (uptake OR use OR utilization OR adoption) AND (East Africa OR West Africa): Filter: Full text online, and from 2015/1/1 - 2025/10/12[Date - Publication] | **415** |
| **Google Scholar** | ("immediate postpartum" OR postnatal OR postpartum OR “Extended postpartum”) AND ("long-acting reversible contraceptive" OR LARC OR implant OR IUD OR IUCD OR Implanon OR Jadelle OR "family planning services" OR "maternal health" OR "primary health care") AND (uptake OR use OR utilization OR adoption) AND (Africa) | **335** |
| Other |  | **2** |
|  | Total | **987** |

**Cochrane library**

**Date Run: 13/12/2025 20:36:02(Rechecked)**

| **ID** | **Search** | **Result** |
| --- | --- | --- |
| #1 | MeSH descriptor: [Postoperative Period] explode all trees | 7460 |
| #2 | Postpartum | 17003 |
| #3 | "Immediate postpartum" | 423 |
| #4 | Postnatal | 8238 |
| #5 | puerperium | 2666 |
| #6 | Extended postpartum | 354 |
| #7 | After childbirth | 4931 |
| #8 | #1 OR #2 OR #3 OR #4 OR #5 OR #5 OR #6 OR #7 | 32908 |
| #9 | MeSH descriptor: [Long-Acting Reversible Contraception] explode all tree | 51 |
| #10 | LARC | 619 |
| #11 | Intrauterine device | 2982 |
| #12 | IUD | 2781 |
| #13 | IUCD | 124 |
| #14 | Implant | 19060 |
| #7 | Contraceptive implant 667 | 360 |
| #16 | Jadelle | 36 |
| #17 | Implanon | 106 |
| #18 | Family planning services | 1941 |
| #9 | Postpartum Family planning | 1 |
| #20 | MeSH descriptor: [Family Planning Services] explode all trees | 445 |
| #21 | #9 OR #10 OR #11 OR #12 OR #13 OR #14 OR #15 OR #16 OR #17 OR #18 OR #19 OR #20 | 25358 |
| #22 | Uptake 26035 |  |
| #23 | Use | 634512 |
| #24 | Utilization | 26255 |
| #25 | Adoption | 7226 |
| #26 | Service uptake | 2321 |
| #27 | Contraceptive uptake | 413 |
| #28 | Contraceptive adoption | 55 |
| #29 | Contraceptive use | 6255 |
| #30 | #22 OR #23 OR #24 OR #25 OR #26 OR #27 OR #28 OR #29 | 668237 |
| #31 | Africa | 14337 |
| #32 | MeSH descriptor: [Africa] explode all trees 13105 |  |
| #33 | #31 OR #32 | 23048 |
| **#34** | **#8 AND #21 AND #30 AND #33 with Cochrane Library publication date Between Jan 2015 and Nov 2025, in Cochrane Reviews** | **98** |
