# Supplementary material for: Uptake and determinants of immediate and extended postpartum long-acting reversible contraceptive use in Eastern and Western Africa: A systematic review and meta-analysis
Source: PLoS One. 2026 Apr 17;21(4):e0346885. doi: 10.1371/journal.pone.0346885 (PMC13089893; doi:10.1371/journal.pone.0346885)
Supplement: S2 File — (DOCX) [file pone.0346885.s002.docx]

**S2 file. JBI cross-sectional study Critical appraisal**

| Study | Q1 | | Q2 | | Q3 | | Q4 | | Q5 | | Q6 | | Q7 | | Q8 | | Quality  Score |
| --- | --- | --- | --- | --- | --- | --- | --- | --- | --- | --- | --- | --- | --- | --- | --- | --- | --- |
|  | A1 | A2 | A1 | A2 | A1 | A2 | A1 | A2 | A1 | A2 | A1 | A2 | A1 | A2 | A1 | A2 |  |
| 1. Areno et al. | Y | Y | Y | Y | P | P | Y | Y | P | P | Y | Y | Y | Y | Y | Y | 7 |
| 1. Tariku et al. | Y | Y | Y | Y | P | P | Y | Y | P | P | Y | Y | Y | Y | Y | Y | 7 |
| 1. Usso et al. | Y | Y | Y | Y | Y | Y | Y | Y | P | P | Y | Y | Y | Y | Y | Y | 7.5 |
| 1. Woldu et al. | Y | Y | Y | Y | P | P | Y | Y | P | P | Y | Y | Y | Y | Y | Y | 7 |
| 1. Wudineh et al. | Y | Y | Y | Y | P | P | Y | Y | P | P | Y | Y | Y | Y | Y | Y | 7 |
| 1. Teshome et al. | Y | Y | Y | Y | Y | Y | Y | Y | P | P | Y | Y | Y | Y | Y | Y | 7.5 |
| 1. Belayihun et al. | Y | Y | Y | Y | Y | Y | Y | Y | P | P | Y | Y | Y | Y | Y | Y | 7.5 |
| 1. Melkie et al. | Y | Y | Y | Y | Y | Y | Y | Y | P | P | Y | Y | Y | Y | Y | Y | 7.5 |
| 1. Abdullahi et al. | Y | Y | Y | Y | Y | Y | Y | Y | P | P | Y | Y | Y | Y | Y | Y | 7.5 |
| 1. Hagos et al. | Y | Y | Y | Y | Y | Y | P | P | P | P | Y | P | Y | P | Y | P | 6.25 |
| 1. Gudeta et al. | Y | Y | Y | Y | Y | Y | Y | Y | Y | P | Y | Y | Y | Y | Y | Y | 7.75 |
| 1. Aemro et al | Y | Y | Y | Y | Y | Y | Y | Y | P | P | Y | Y | Y | Y | Y | Y | 7.5 |
| 1. Geda et al. | Y | Y | Y | Y | Y | Y | Y | Y | P | P | Y | Y | Y | Y | Y | Y | 7.5 |
| 1. Guye et al. | Y | Y | Y | Y | Y | Y | Y | Y | P | P | Y | Y | Y | Y | Y | Y | 7.5 |
| 1. Ayane et al. | Y | Y | Y | Y | Y | Y | Y | Y | Y | Y | Y | Y | Y | Y | Y | Y | 8 |
| 1. Asnake et al. | Y | Y | Y | Y | Y | Y | Y | Y | Y | Y | Y | Y | Y | Y | Y | Y | 8 |
| 1. Demissie et al. | Y | Y | Y | Y | Y | Y | Y | Y | P | P | Y | Y | Y | Y | Y | Y | 7.5 |
| 1. Dinsa et al. | Y | Y | Y | Y | Y | Y | Y | Y | P | P | Y | Y | Y | Y | Y | Y | 7.5 |
| 1. Tefera et al. | Y | Y | Y | Y | Y | Y | Y | Y | P | P | Y | Y | Y | Y | Y | Y | 7.5 |
| 1. Kitessa et al | Y | Y | Y | Y | Y | Y | Y | Y | P | P | Y | Y | Y | Y | Y | Y | 7.5 |
| 1. Feleke | Y | Y | Y | Y | Y | Y | Y | Y | Y | Y | Y | Y | Y | Y | Y | Y | 8 |
| 1. Shiferaw et al. | Y | Y | Y | Y | Y | Y | Y | Y | Y | Y | Y | Y | Y | Y | Y | Y | 8 |
| 1. Silesh et al. | Y | Y | Y | Y | Y | Y | Y | Y | Y | Y | Y | Y | Y | Y | Y | Y | 8 |
| 1. Tesfaye et al. | Y | Y | Y | Y | Y | Y | Y | Y | P | P | Y | Y | Y | Y | Y | Y | 7.5 |
| 1. Tegene et al. | Y | Y | Y | Y | Y | Y | Y | Y | P | P | Y | Y | Y | Y | Y | Y | 7.5 |
| 1. Mesfin & Wallellgn | Y | Y | Y | Y | Y | Y | Y | Y | N | N | Y | Y | Y | Y | Y | Y | 7 |
| 1. Tamire et al. | Y | Y | Y | Y | Y | Y | Y | Y | Y | Y | Y | Y | Y | Y | Y | Y | 8 |
| 1. Eristu et al. | Y | Y | Y | Y | Y | Y | Y | Y | Y | Y | Y | Y | Y | Y | Y | Y | 8 |
| 1. Anguzu et al | Y | Y | Y | Y | Y | Y | Y | Y | Y | Y | Y | Y | Y | Y | Y | Y | 8 |
| 1. Gebremedhin et al. | Y | Y | Y | Y | Y | Y | Y | Y | P | P | Y | Y | Y | Y | Y | Y | 7.5 |
| 1. Aliyi | Y | Y | Y | Y | Y | Y | Y | Y | P | P | Y | Y | Y | Y | Y | Y | 8 |
| 1. Andualem et al. | Y | Y | Y | Y | Y | Y | Y | Y | Y | Y | Y | Y | Y | Y | Y | Y | 8 |
| 1. Appiah et al. | Y | Y | Y | Y | Y | Y | Y | Y | P | N | Y | Y | Y | Y | Y | Y | 7.25 |
| 1. Nakiwunga et al. | Y | Y | Y | Y | Y | Y | Y | Y | P | N | Y | Y | Y | Y | Y | Y | 7.25 |
| 1. Shabiby et al. | Y | Y | Y | Y | Y | Y | Y | Y | P | N | Y | Y | Y | Y | Y | Y | 7.25 |
| 1. Mihretie et al. | Y | Y | Y | Y | Y | Y | Y | Y | P | N | Y | Y | Y | Y | Y | Y | 7.25 |
| 1. Abebe et al. | Y | Y | Y | Y | Y | Y | Y | Y | P | P | Y | Y | Y | Y | Y | Y | 7.25 |
| 1. Abraha et al. (2018) | Y | Y | Y | Y | Y | Y | Y | Y | P | N | Y | Y | Y | Y | Y | Y | 7.25 |
| 1. Agula et al. | Y | Y | Y |  | Y | Y | Y | Y | P | N | Y | Y | Y | Y | Y | Y | 7.25 |
| 1. Alupo et al. | Y | Y | Y |  | Y | Y | Y | Y | P | P | Y | Y | Y | Y | Y | Y | 7.5 |
| 1. Jaleta et al. | Y | Y | Y |  | Y | Y | Y | Y | P | N | Y | Y | Y | Y | Y | Y | 7.25 |
| 1. Gadigbe et al. | Y | Y | Y |  | Y | Y | Y | Y | P | N | Y | Y | Y | Y | Y | Y | 7.25 |
| 1. Getaneh et al. | Y | Y | Y |  | Y | Y | Y | Y | P | P | Y | Y | Y | Y | Y | Y | 7.5 |
| 1. Gebremedhin et al | Y | Y | Y |  | Y | Y | Y | Y | P | N | Y | Y | Y | Y | Y | Y | 7.25 |
| 1. Kachiro et al. | Y | Y | Y |  | Y | Y | Y | Y | P | N | Y | Y | Y | Y | Y | Y | 7.25 |
| 1. Kanakuze et al. | Y | Y | Y |  | Y | Y | Y | Y | P | N | Y | Y | Y | Y | Y | Y | 7.25 |
| 1. Kenate & Amenu | Y | Y | Y | Y | Y | Y | Y | Y | P | p | N | Y | Y | Y | Y | Y | 7.25 |
| 1. Mogeni et al. | Y | Y | Y | Y | Y | Y | Y | Y | Y | Y | Y | Y | Y | Y | Y | Y | 8 |
| 1. Niguse et al. | Y | Y | Y | Y | Y | Y | Y | Y | Y | Y | Y | Y | Y | Y | Y | Y | 8 |
| 1. Nugussa et al. | Y | Y | Y | Y | Y | Y | Y | Y | Y | Y | Y | Y | Y | Y | Y | Y | 8 |
| 1. Obua et al. | Y | Y | Y | Y | P | Y | Y | Y | P | N | Y | Y | Y | Y | Y | Y | 7.25 |
| 1. Omona & Namuli | Y | Y | Y | Y | Y | Y | Y | Y | Y | Y | Y | Y | Y | Y | Y | Y | 8 |
| 1. Tafa & Worku | Y | Y | Y | Y | Y | Y | Y | Y | Y | Y | Y | Y | Y | Y | Y | Y | 8 |
| 1. Bizuneh | Y | Y | Y | Y | Y | Y | Y | Y | Y | Y | Y | Y | Y | Y | Y | Y | 8 |
| 1. Negash | Y | Y | Y | Y | Y | Y | Y | Y | Y | Y | Y | Y | Y | Y | Y | Y | 8 |
| 1. Ashebir et al. | Y | Y | Y | Y | Y | Y | Y | Y | Y | Y | Y | Y | Y | Y | Y | Y | 8 |
| 1. Nugussie et al. | Y | Y | Y | Y | Y | Y | Y | Y | Y | Y | Y | Y | Y | Y | Y | Y | 8 |
| 1. Asah‑Opoku et al | Y | Y | Y | P | P | Y | Y | Y | P | P | Y | Y | Y | Y | Y | Y | 7 |
| 1. Mengesha et al. | Y | Y | Y | Y | Y | Y | Y | Y | Y | Y | Y | Y | Y | Y | Y | Y | 8 |
| 1. Wekere et al. | Y | Y | Y | Y | Y | Y | Y | Y | P | P | Y | Y | Y | Y | Y | Y | 7.5 |
| 1. Gejo et al. | Y | Y | Y | Y | Y | Y | Y | Y | P | P | Y | Y | Y | Y | Y | Y | 7.5 |
| 1. Abraha et al. (2017) | Y | Y | Y | Y | Y | Y | Y | Y | Y | Y | Y | Y | Y | Y | Y | Y | 8 |
| 1. Assefa et al. | Y | Y | Y | Y | Y | Y | Y | Y | Y | Y | Y | Y | Y | Y | Y | Y | 8 |

*A1: Assessor one; A2: Assessor two; Q1: Clarity of Objectives Q2: Study Design Appropriateness Q3: Sampling and Participants Q4: Data Collection Methods Q5: Bias and Confounding Control Q6: Statistical Analysis Q7: Results Transparency Q8: Conclusions and Relevance*

**JBI Quasi‑Experimental appraisal table**

| **Author** | Q1 | | Q2 | | Q3 | | Q4 | | Q5 | | Q6 | | Q7 | | Q8 | | Q9 | | **Quality**  **score** |
| --- | --- | --- | --- | --- | --- | --- | --- | --- | --- | --- | --- | --- | --- | --- | --- | --- | --- | --- | --- |
| 1. Sium et al. | Y | Y | Y | Y | Y | Y | N | N | Y | Y | Y | Y | Y | Y | Y | Y | Y | Y | 8 |
| 1. Sori et al. | Y | Y | Y | Y | Y | Y | N | N | Y | Y | Y | Y | Y | Y | Y | Y | Y | Y | 8 |

*Q1: Cause/Effect clear Q2: Participants similar Q3: Similar treatment aside from intervention, Q4: Control group, Q5: Multiple measurements pre/post Q6: Follow up complete Q7: Outcomes measured same way Q8: Outcomes measured reliably Q9: Appropriate statistical analysis*

**JBI Case-control Critical appraisal table**

| **Study** | **Q1** | | **Q2** | | **Q3** | | **Q4** | | **Q5** | | **Q6** | | **Q7** | | **Q8** | | **Q9** | | **Q10** | | **Quality**  **score** |
| --- | --- | --- | --- | --- | --- | --- | --- | --- | --- | --- | --- | --- | --- | --- | --- | --- | --- | --- | --- | --- | --- |
| 1. Adella et al. | Y | Y | Y | Y | Y | Y | Y | Y | Y | Y | Y | Y | Y | Y | N | N | Y | Y | Y | Y | 9 |
| 1. Assefaw et al | Y | Y | P | Y | Y | Y | Y | Y | Y | Y | Y | Y | Y | Y | N | N | Y | Y | Y | Y | 8.75 |
| 1. Seid et al. | Y | Y | Y | Y | Y | Y | Y | Y | Y | Y | Y | Y | Y | Y | N | N | Y | Y | Y | Y | 9 |

*Q1: Groups comparable, Q2: Matching appropriate, Q3: Same criteria for cases/controls, Q4: Exposure measured validly, Q5: Exposure measured same way, Q6: Confounders identified, Q7: Confounders addressed, Q8: Outcomes assessed reliably, Q9: Exposure period adequate, Q10: Statistical analysis appropriate*
