# Supplementary material for: Uptake and determinants of immediate and extended postpartum long-acting reversible contraceptive use in Eastern and Western Africa: A systematic review and meta-analysis
Source: PLoS One. 2026 Apr 17;21(4):e0346885. doi: 10.1371/journal.pone.0346885 (PMC13089893; doi:10.1371/journal.pone.0346885)
Supplement: S1 Table — (DOCX) [file pone.0346885.s003.docx]

**S1Table.** Leave-One-Out Meta-Analysis for Pooled Prevalence of IPP-LARC

| Study Omitted | Proportion | 95% CI | I² (%) |
| --- | --- | --- | --- |
| Teshome et al. | 0.2118 | [0.1572;0.2721] | 98.3 |
| Arero et al. | 0.2052 | [0.1541; 0.2614] | 98.0 |
| Belayihun et al. | 0.2113 | [0.1567; 0.2716] | 98.2 |
| Demissie et al. | 22.18 | [16.59; 28.33] | 98.2 |
| Gudeta et al. | 0.2204 | [0.1638; 0.2827] | 98.2 |
| Gebremichael | 0.2194 | [0.1629; 0.2817] | 98.3 |
| Asnake et al. | 0.2084 | [0.1567; 0.2653] | 98.0 |
| Silesh et al. | 0.2176 | [0.1617; 0.2792] | 98.3 |
| Sium et al. | 0.2205 | [0.1638; 0.2829] | 98.2 |
| Sori et al. | 0.2259 | [0.1723; 0.2843] | 98.0 |
| Ayena et al. | 0.2150 | [0.1594; 0.2763] | 98.3 |
| Tariku et al. | 0.2158 | [0.1600; 0.2773] | 98.3 |
| Tegene et al. | 0.2085 | [0.1554; 0.2671] | 98.2 |
| Tesfaye et al. | 0.2113 | [0.1570; 0.2714] | 98.3 |
| Usso et al. | 0.2189 | [0.1627; 0.2809] | 98.3 |
| Gadigbe et al. | 0.2131 | [0.1573; 0.2748] | 98.2 |
| Kitessa et al. | 0.2246 | [0.1692; 0.2854] | 98.3 |
| Bizuneh | 0.2124 | [0.1576; 0.2729] | 98.3 |
| Nakiwunga et al. | 0.2220 | [0.1663; 0.2832] | 98.2 |
| Wudineh et al. | 0.2136 | [0.1583; 0.2746] | 98.3 |
| Melkie et al. | 0.2238 | [0.1684; 0.2845] | 98.2 |
| Kachiro et al. | 0.2300 | [0.1749; 0.2901] | 98.2 |
| Obua et al. | 0.2303 | [0.1768; 0.2886] | 98.0 |
| Combined | **21.74** | **[16.36, 27.65]** | **98.2** |
