# Supplementary material for: Uptake and determinants of immediate and extended postpartum long-acting reversible contraceptive use in Eastern and Western Africa: A systematic review and meta-analysis
Source: PLoS One. 2026 Apr 17;21(4):e0346885. doi: 10.1371/journal.pone.0346885 (PMC13089893; doi:10.1371/journal.pone.0346885)
Supplement: S2 Table — (DOCX) [file pone.0346885.s004.docx]

**S2 Table.** Leave-One-Out Meta-Analysis for Pooled Prevalence of IPP-I

| Study Omitted | Proportion | 95% CI | I² (%) |
| --- | --- | --- | --- |
| Teshome et al. | 0.2005 | [0.1469; 0.2599] | 98.2 |
| Arero et al. | 0.1936 | [0.1438; 0.2488] | 98.0 |
| Belayihun et al. | 0.2097 | [0.1542; 0.2713] | 98.2 |
| Demissie et al. | 0.2133 | [0.1593; 0.2727] | 98.1 |
| Gudeta et al. | 0.2073 | [0.1513; 0.2695] | 98.2 |
| Asnake et al. | 0.1993 | [0.1464; 0.2580] | 98.1 |
| Silesh et al. | 0.2063 | [0.1514; 0.2673] | 98.3 |
| Sium et al. | 0.2085 | [0.1526; 0.2706] | 98.2 |
| Sori et al. | 0.2139 | [0.1607; 0.2723] | 98.0 |
| Ayena et al. | 0.2039 | [0.1493; 0.2645] | 98.2 |
| Tariku et al. | 0.2053 | [0.1504; 0.2662] | 98.3 |
| Tegene et al. | 0.1957 | [0.1446; 0.2524] | 98.1 |
| Tesfaye et al. | 0.2030 | [0.1490; 0.2630] | 98.2 |
| Usso et al. | 0.2088 | [0.1535; 0.2700] | 98.2 |
| Gadigbe et al. | 0.2041 | [0.1485; 0.2660] | 98.2 |
| Mogeni et al. | 0.1949 | [0.1440; 0.2514] | 98.1 |
| Bizuneh | 0.2002 | [0.1468; 0.2596] | 98.2 |
| Shabiby et al. | 0.1930 | [0.1421; 0.2496] | 98.1 |
| Nakiwunga et al. | 0.2089 | [0.1539; 0.2697] | 98.2 |
| Wudineh et al. | 0.2035 | [0.1489; 0.2641] | 98.2 |
| Melkie et al. | 0.2136 | [0.1595; 0.2732] | 98.1 |
| Obua et al. | 0.2181 | [0.1652; 0.2761] | 98.0 |
| Combined | **0.2047** | **[0.1521; 0.2629],** | **98.2%** |
