# Supplementary material for: Uptake and determinants of immediate and extended postpartum long-acting reversible contraceptive use in Eastern and Western Africa: A systematic review and meta-analysis
Source: PLoS One. 2026 Apr 17;21(4):e0346885. doi: 10.1371/journal.pone.0346885 (PMC13089893; doi:10.1371/journal.pone.0346885)
Supplement: S3 Table — (DOCX) [file pone.0346885.s005.docx]

**S3 Table.** Leave-One-Out Meta-Analysis for Pooled Prevalence of IPP-IUD

| Study Omitted | Proportion | 95% CI | I² (%) |
| --- | --- | --- | --- |
| Teshome et al. | 0.0746 | [0.0500; 0.1034] | 97.1 |
| Arero et al. | 0.0742 | [0.0497; 0.1032] | 97.1 |
| Belayihun et al. | 0.0764 | [0.0518; 0.1052] | 97.0 |
| Demissie et al. | 0.0752 | [0.0504; 0.1043] | 97.1 |
| Silesh et al. | 0.0757 | [0.0510; 0.1046] | 97.1 |
| Sium et al. | 0.0762 | [0.0516; 0.1051] | 97.0 |
| Sori et al. | 0.0766 | [0.0521; 0.1053] | 96.9 |
| Ayena et al. | 0.0747 | [0.0500; 0.1036] | 97.1 |
| Tariku et al. | 0.0742 | [0.0496; 0.1031] | 97.1 |
| Tegene et al. | 0.0762 | [0.0516; 0.1051] | 97.1 |
| Tesfaye et al. | 0.0721 | [0.0482; 0.1004] | 97.1 |
| Usso et al. | 0.0743 | [0.0497; 0.1034] | 97.1 |
| Gadigbe et al. | 0.0729 | [0.0483; 0.1019] | 97.1 |
| Bizuneh | 0.0756 | [0.0510; 0.1045] | 97.1 |
| Wudineh et al. | 0.0737 | [0.0492; 0.1025] | 97.1 |
| Melkie et al. | 0.0750 | [0.0504; 0.1040] | 97.1 |
| Abdullahi et al. | 0.0725 | [0.0483; 0.1010] | 97.1 |
| Aemro et al. | 0.0697 | [0.0470; 0.0962] | 96.8 |
| Dinsa et al. | 0.0704 | [0.0471; 0.0979] | 97.0 |
| Geda et al. | 0.0715 | [0.0477; 0.0995] | 97.1 |
| Guye et al. | 0.0685 | [0.0473; 0.0932] | 96.3 |
| Hagos et al. | 0.0753 | [0.0508; 0.1041] | 97.1 |
| Tefera et al. | 0.0698 | [0.0468; 0.0969] | 96.9 |
| Gebremedhin et al. | 0.0717 | [0.0478; 0.0999] | 97.1 |
| Kanakuze et al. | 0.0684 | [0.0465; 0.0941] | 96.7 |
| Shiferaw et al. | 0.0728 | [0.0486; 0.1014] | 97.1 |
| Alupo et al. | 0.0759 | [0.0513; 0.1048] | 97.1 |
| Obua et al. | 0.0772 | [0.0527; 0.1058] | 97.0 |
| Gudeta et al. | 0.0758 | [0.0509; 0.1049] | 97.1 |
| Combined | **0.0737** | **[0.0499; 0.1015],** | **97.0%** |
