# Supplementary material for: Uptake and determinants of immediate and extended postpartum long-acting reversible contraceptive use in Eastern and Western Africa: A systematic review and meta-analysis
Source: PLoS One. 2026 Apr 17;21(4):e0346885. doi: 10.1371/journal.pone.0346885 (PMC13089893; doi:10.1371/journal.pone.0346885)
Supplement: S4 Table — (DOCX) [file pone.0346885.s006.docx]

**S4 Table.** Leave-One-Out Meta-Analysis for Pooled Prevalence of EPP-LARC

| Study Omitted | Proportion | 95% CI | I² |
| --- | --- | --- | --- |
| Eristu et al. (2024) | 0.1884 | [0.1271; 0.2584] | 98.9% |
| Mesfin et al. (2021) | 0.1934 | [0.1306; 0.2650] | 98.9% |
| Tamrie et al. (2015) | 0.1883 | [0.1271; 0.2582] | 98.9% |
| Woldu et al. (2020) | 0.1884 | [0.1272; 0.2583] | 98.9% |
| Agula et al. (2022) | 0.1934 | [0.1301; 0.2656] | 98.9% |
| Jaleta et al. (2024) | 0.1959 | [0.1327; 0.2678] | 98.9% |
| Getaneh et al. (2021) | 0.2017 | [0.1388; 0.2730] | 98.9% |
| Kenate & Amenu (2015) | 0.1985 | [0.1358; 0.2696] | 98.9% |
| Niguse et al. (2019) | 0.2051 | [0.1433; 0.2748] | 98.8% |
| Nugussa et al. (2023) | 0.1892 | [0.1277; 0.2595] | 98.9% |
| Tafa & Worku (2018) | 0.1897 | [0.1278; 0.2605] | 98.9% |
| Gebremedhin et al. (2018) | 0.1915 | [0.1285; 0.2635] | 98.9% |
| Aliyi (2017) | 0.2008 | [0.1375; 0.2725] | 98.9% |
| Andualem et al. (2022) | 0.1930 | [0.1304; 0.2645] | 98.9% |
| Appiah et al. (2024) | 0.2031 | [0.1404; 0.2740] | 98.9% |
| Mihretie et al. (2020) | 0.2003 | [0.1372; 0.2719] | 98.9% |
| Negash (2020) | 0.1894 | [0.1277; 0.2601] | 98.9% |
| Anguzu et al. (2018) | 0.2000 | [0.1368; 0.2716] | 98.9% |
| Nigussie et al. (2016) | 0.2005 | [0.1373; 0.2722] | 98.9% |
| Abebe et al. (2023) | 0.1973 | [0.1341; 0.2691] | 98.9% |
| Assefa et al. (2021) | 0.1914 | [0.1291; 0.2625] | 98.9% |
| Abraha et al. (2018) | 0.1951 | [0.1310; 0.2685] | 98.9% |
| Gejo et al. (2019) | 0.1902 | [0.1284; 0.2608] | 98.9% |
| Wekere et al. (2019) | 0.1773 | [0.1269; 0.2341] | 98.4% |
| Mengesha et al. (2015) | 0.2052 | [0.1450; 0.2728] | 98.7% |
| Combined | **0.1946** | **[0.1338; 0.2637]** | **98.9%** |
