# Supplementary material for: Uptake and determinants of immediate and extended postpartum long-acting reversible contraceptive use in Eastern and Western Africa: A systematic review and meta-analysis
Source: PLoS One. 2026 Apr 17;21(4):e0346885. doi: 10.1371/journal.pone.0346885 (PMC13089893; doi:10.1371/journal.pone.0346885)
Supplement: S5 Table — (DOCX) [file pone.0346885.s007.docx]

**S5 Table.** Leave-One-Out Meta-Analysis for Pooled Prevalence of EPP-I

| Study Omitted | Proportion | 95% CI | I² |
| --- | --- | --- | --- |
| Abraha et al. | 0.1638 | [0.1061; 0.2309] | 99.0% |
| Eristu et al. | 0.1570 | [0.1013; 0.2221] | 99.0% |
| Mesfin et al. | 0.1611 | [0.1042; 0.2275] | 99.0% |
| Tamrie et al. | 0.1538 | [0.0997; 0.2169] | 99.0% |
| Woldu et al. | 0.1561 | [0.1008; 0.2207] | 99.0% |
| Abebe et al. | 0.1638 | [0.1066; 0.2305] | 99.0% |
| Agula et al. | 0.1616 | [0.1042; 0.2287] | 99.0% |
| Jaleta et al. | 0.1630 | [0.1058; 0.2298] | 99.0% |
| Getaneh et al. | 0.1669 | [0.1093; 0.2337] | 99.0% |
| Kenate & Amenu | 0.1661 | [0.1091; 0.2323] | 99.0% |
| Niguse et al. | 0.1702 | [0.1133; 0.2359] | 99.0% |
| Nugussa et al. | 0.1601 | [0.1035; 0.2262] | 99.0% |
| Tafa & Worku | 0.1603 | [0.1033; 0.2271] | 99.0% |
| Gebremedhin et al. | 0.1659 | [0.1080; 0.2333] | 99.0% |
| Aliyi | 0.1668 | [0.1091; 0.2339] | 99.0% |
| Andualem et al. | 0.1608 | [0.1040; 0.2272] | 99.0% |
| Mihretie et al. | 0.1667 | [0.1093; 0.2334] | 99.0% |
| Negash | 0.1570 | [0.1013; 0.2222] | 99.0% |
| Anguzu et al. | 0.1663 | [0.1089; 0.2330] | 99.0% |
| Ashebir et al. | 0.1668 | [0.1092; 0.2338] | 99.0% |
| Nigussie et al. | 0.1675 | [0.1101; 0.2341] | 99.0% |
| Gejo et al. | 0.1591 | [0.1028; 0.2248] | 99.0% |
| Assefa et al. | 0.1602 | [0.1035; 0.2263] | 99.0% |
| Asah-Opoku et al. | 0.1461 | [0.0999; 0.1994] | 98.6% |
| Wekere et al. | 0.1519 | [0.0997; 0.2127] | 98.9% |
| Abraham et al. (2017) | 0.1629 | [0.1048; 0.2309] | 99.0% |
| Mengesha et al. | 0.1725 | [0.1181; 0.2347] | 98.8% |
| Combined | **0.1620** | **[0.1067; 0.2262]** | **99.0%** |
