# Supplementary material for: Uptake and determinants of immediate and extended postpartum long-acting reversible contraceptive use in Eastern and Western Africa: A systematic review and meta-analysis
Source: PLoS One. 2026 Apr 17;21(4):e0346885. doi: 10.1371/journal.pone.0346885 (PMC13089893; doi:10.1371/journal.pone.0346885)
Supplement: S6 Table — (DOCX) [file pone.0346885.s008.docx]

**S6 Table.** Leave-One-Out Meta-Analysis for Pooled Prevalence of EPPIUCD

| Study Omitted | Proportion | 95% CI | I² |
| --- | --- | --- | --- |
| Eristu et al. | 0.0446 | [0.0289; 0.0635] | 95.1% |
| Mesfin et al. | 0.0451 | [0.0294; 0.0639] | 95.1% |
| Tamrie et al. | 0.0456 | [0.0298; 0.0644] | 95.1% |
| Woldu et al. | 0.0460 | [0.0302; 0.0648] | 95.1% |
| Abebe et al. | 0.0463 | [0.0305; 0.0651] | 95.0% |
| Agula et al. | 0.0445 | [0.0288; 0.0634] | 95.1% |
| Jaleta et al. | 0.0456 | [0.0298; 0.0644] | 95.1% |
| Nigussie et al. | 0.0457 | [0.0298; 0.0646] | 95.1% |
| Kenate & Amenu | 0.0451 | [0.0296; 0.0635] | 95.1% |
| Niguse et al. | 0.0476 | [0.0321; 0.0660] | 94.6% |
| Nugussa et al. | 0.0422 | [0.0277; 0.0596] | 94.6% |
| Omona & Namuli | 0.0416 | [0.0272; 0.0587] | 94.7% |
| Tafa & Worku | 0.0424 | [0.0279; 0.0596] | 94.4% |
| Gebremedhin et al. | 0.0463 | [0.0303; 0.0653] | 94.9% |
| Aliyi | 0.0467 | [0.0309; 0.0655] | 94.8% |
| Andualem et al. | 0.0450 | [0.0293; 0.0638] | 95.1% |
| Mihretie et al. | 0.0462 | [0.0304; 0.0650] | 95.0% |
| Negash | 0.0460 | [0.0302; 0.0650] | 95.0% |
| Anguzu et al. | 0.0464 | [0.0306; 0.0652] | 95.0% |
| Assefa et al. | 0.0441 | [0.0286; 0.0625] | 95.1% |
| Abraha et al. | 0.0450 | [0.0290; 0.0642] | 95.1% |
| Wekere et al. | 0.0399 | [0.0282; 0.0535] | 91.6% |
| Gejo et al. | 0.0440 | [0.0286; 0.0625] | 95.1% |
| Mengesha et al. | 0.0464 | [0.0305; 0.0654] | 94.9% |
| Combined | **0.0449** | **[0.0298; 0.0629]** | **94.9%** |
