# Supplementary material for: Uptake and determinants of immediate and extended postpartum long-acting reversible contraceptive use in Eastern and Western Africa: A systematic review and meta-analysis
Source: PLoS One. 2026 Apr 17;21(4):e0346885. doi: 10.1371/journal.pone.0346885 (PMC13089893; doi:10.1371/journal.pone.0346885)
Supplement: S1 Fig — (DOCX) [file pone.0346885.s009.docx]

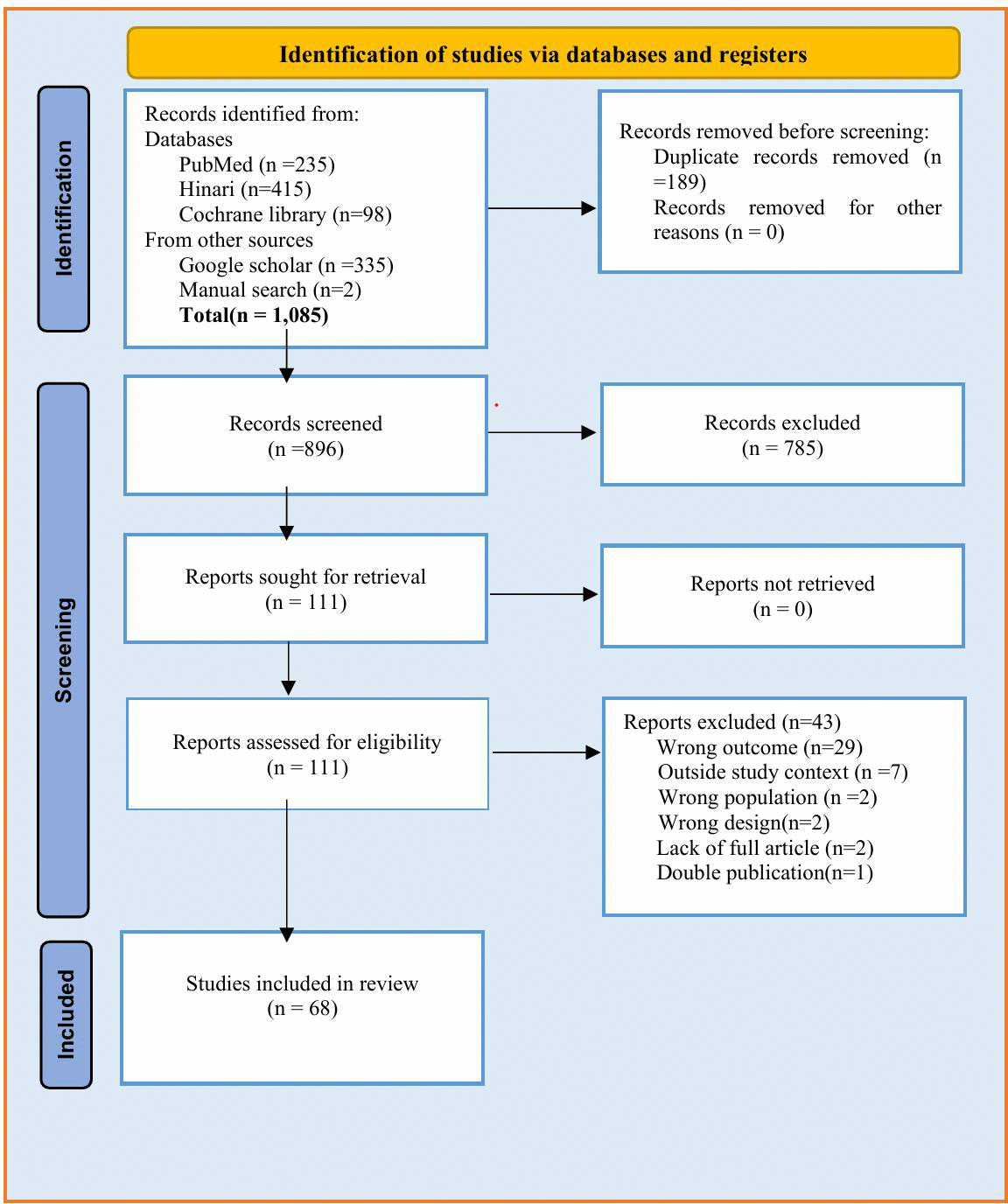


**S1 Fig.** PRISMA flow diagram of study selection for immediate and extended postpartum LARC uptake in Eastern Africa and Western Africa, 2025.(TIF)
