# Supplementary figures and images for: Uptake and determinants of immediate and extended postpartum long-acting reversible contraceptive use in Eastern and Western Africa: A systematic review and meta-analysis
Source: PLoS One. 2026 Apr 17;21(4):e0346885. doi: 10.1371/journal.pone.0346885 (PMC13089893; doi:10.1371/journal.pone.0346885)

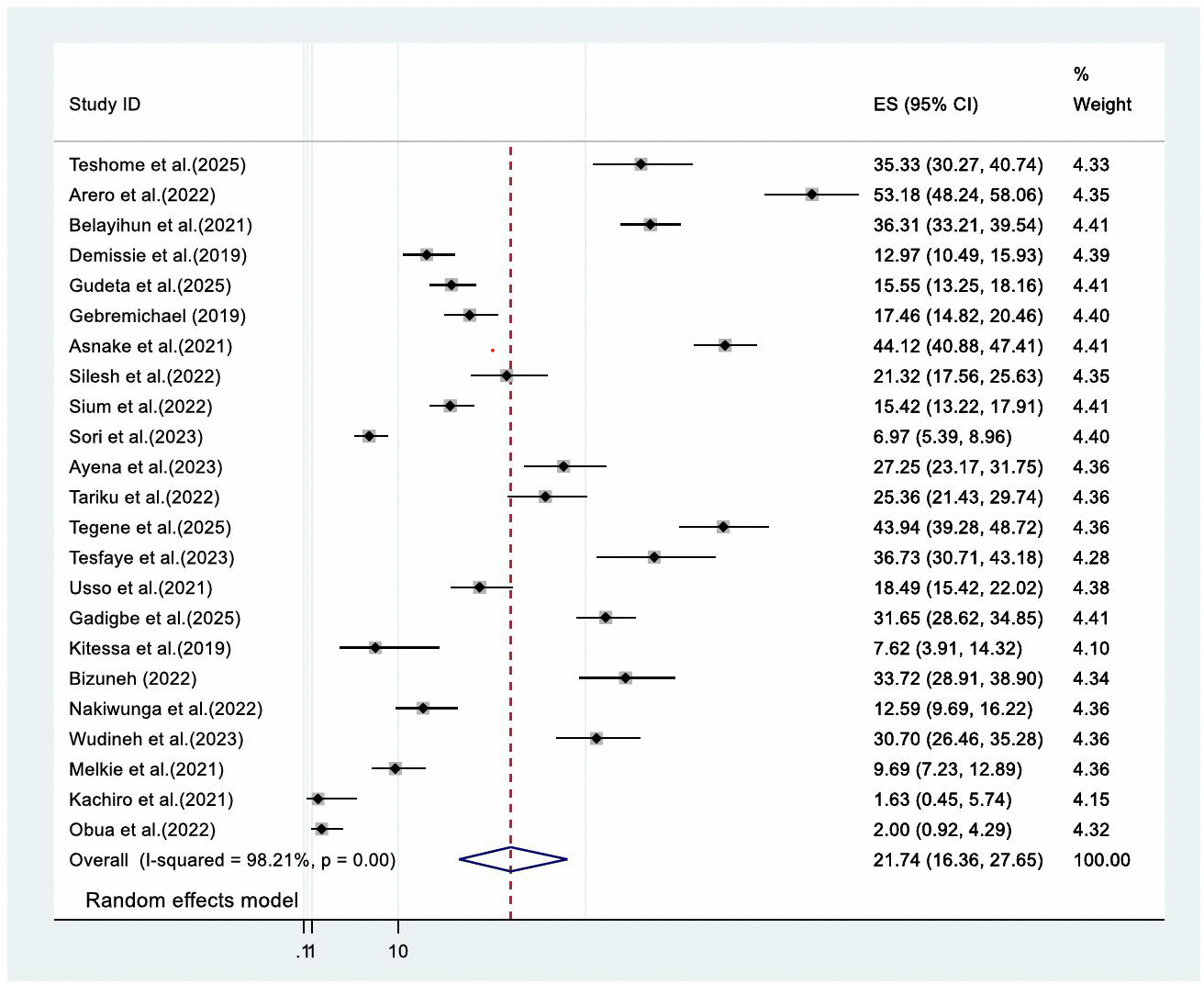


**S2 Fig.** Pooled prevalence of IPP-LARC uptake in Eastern and Western Africa, 2025.(TIF)

Supplement: S2 Fig — (DOCX) [file pone.0346885.s010.docx]

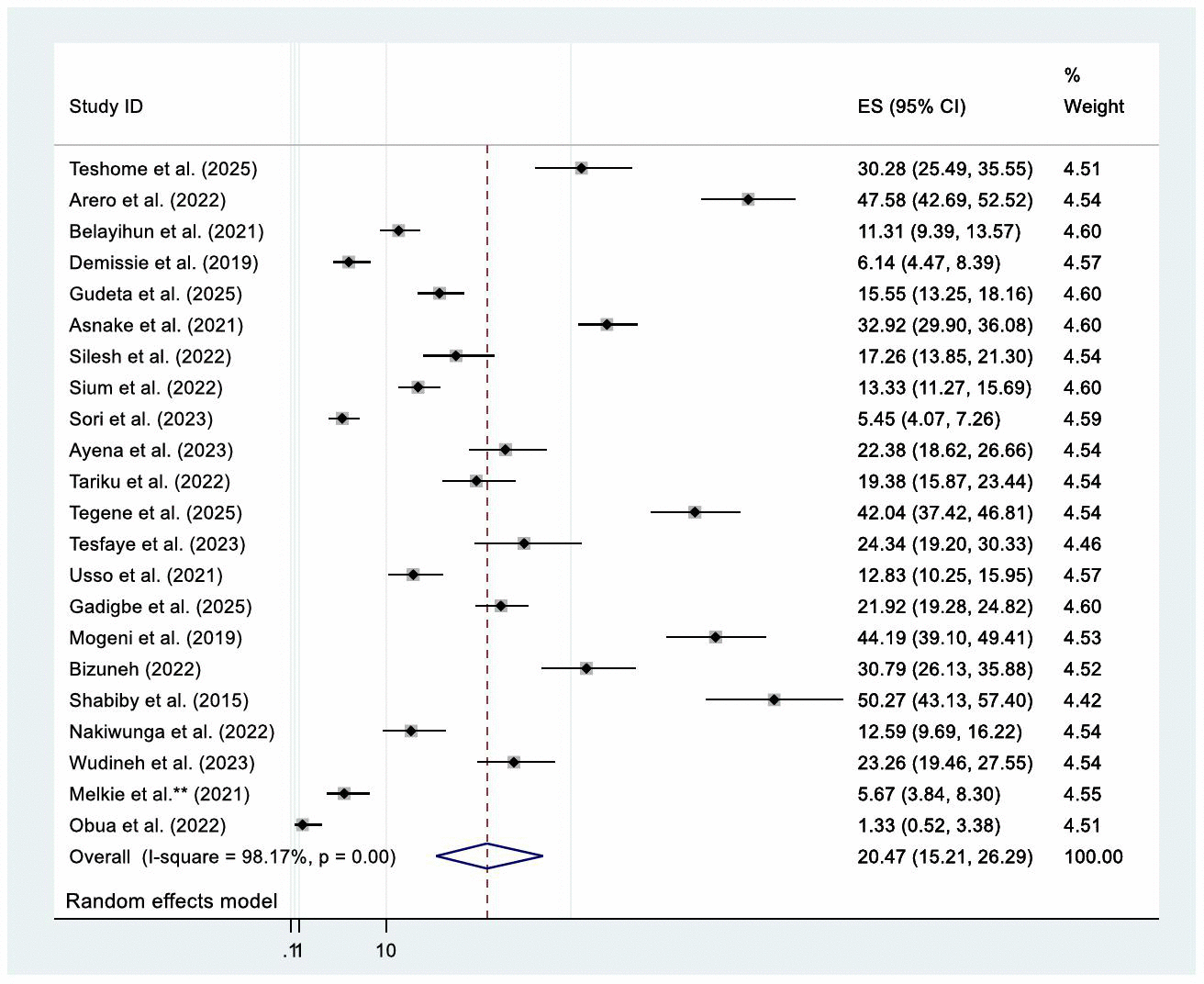


**S3 Fig.**  Pooled prevalence of IPP-I uptake in Eastern and Western Africa, 2025.(TIF)

Supplement: S3 Fig — (DOCX) [file pone.0346885.s011.docx]

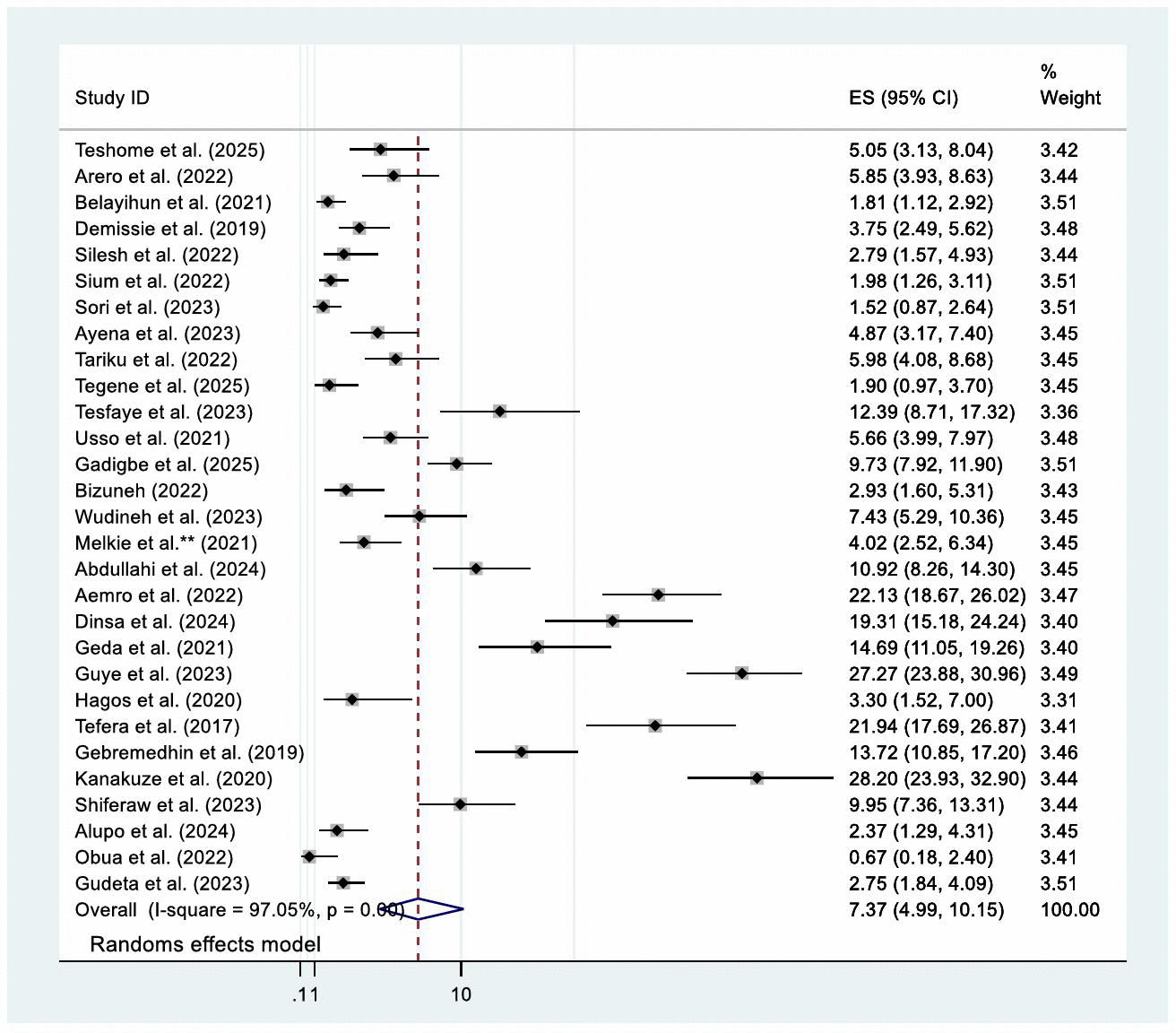
 **S4 Fig.**  Pooled prevalence of IPP-IUD uptake in Eastern and Western Africa, 2025.(TIF)

Supplement: S4 Fig — (DOCX) [file pone.0346885.s012.docx]

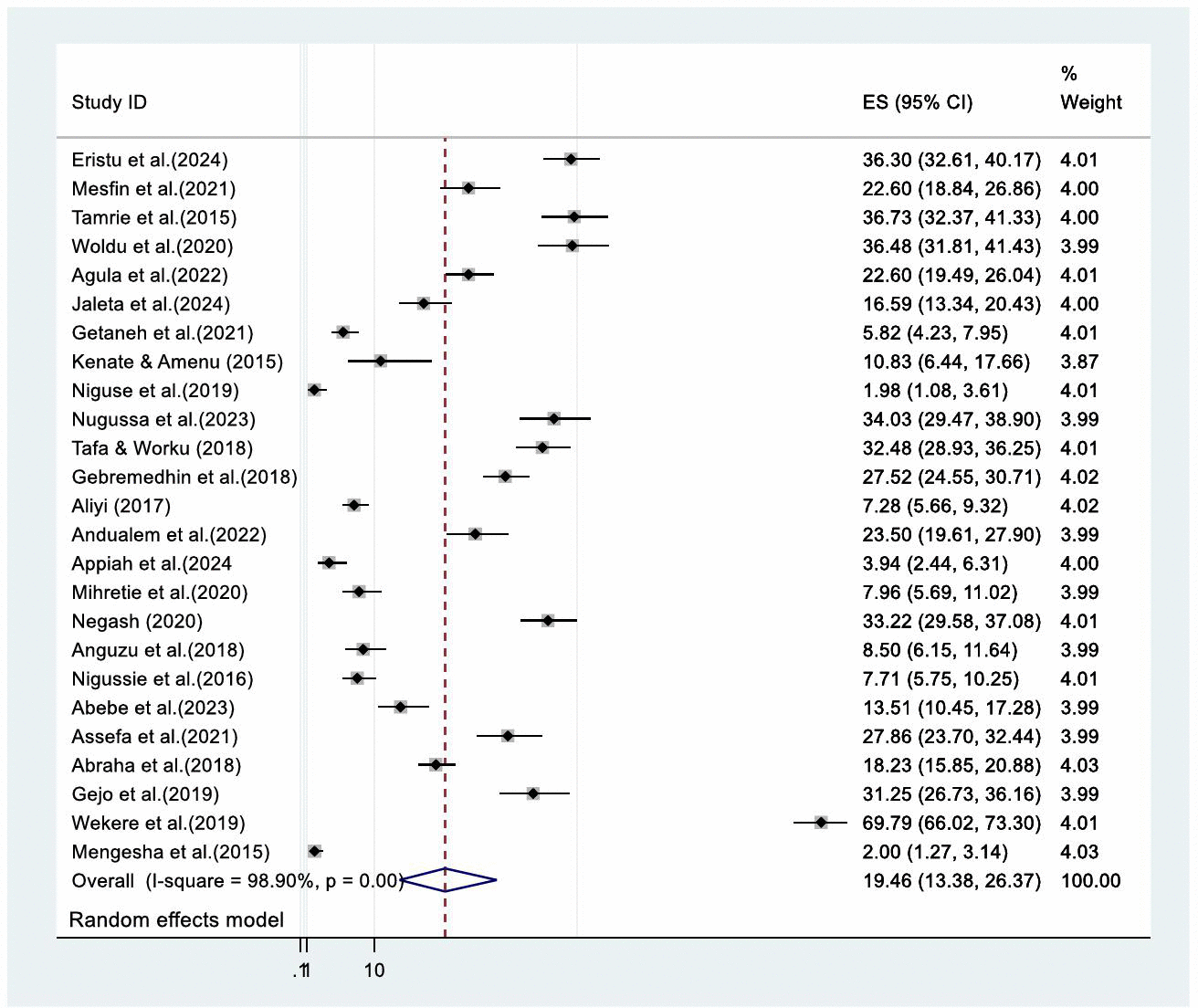
 **S5 Fig.** Pooled prevalence of EPP-LARC uptake in Eastern and Western Africa, 2025.(TIF)

Supplement: S5 Fig — (DOCX) [file pone.0346885.s013.docx]

*
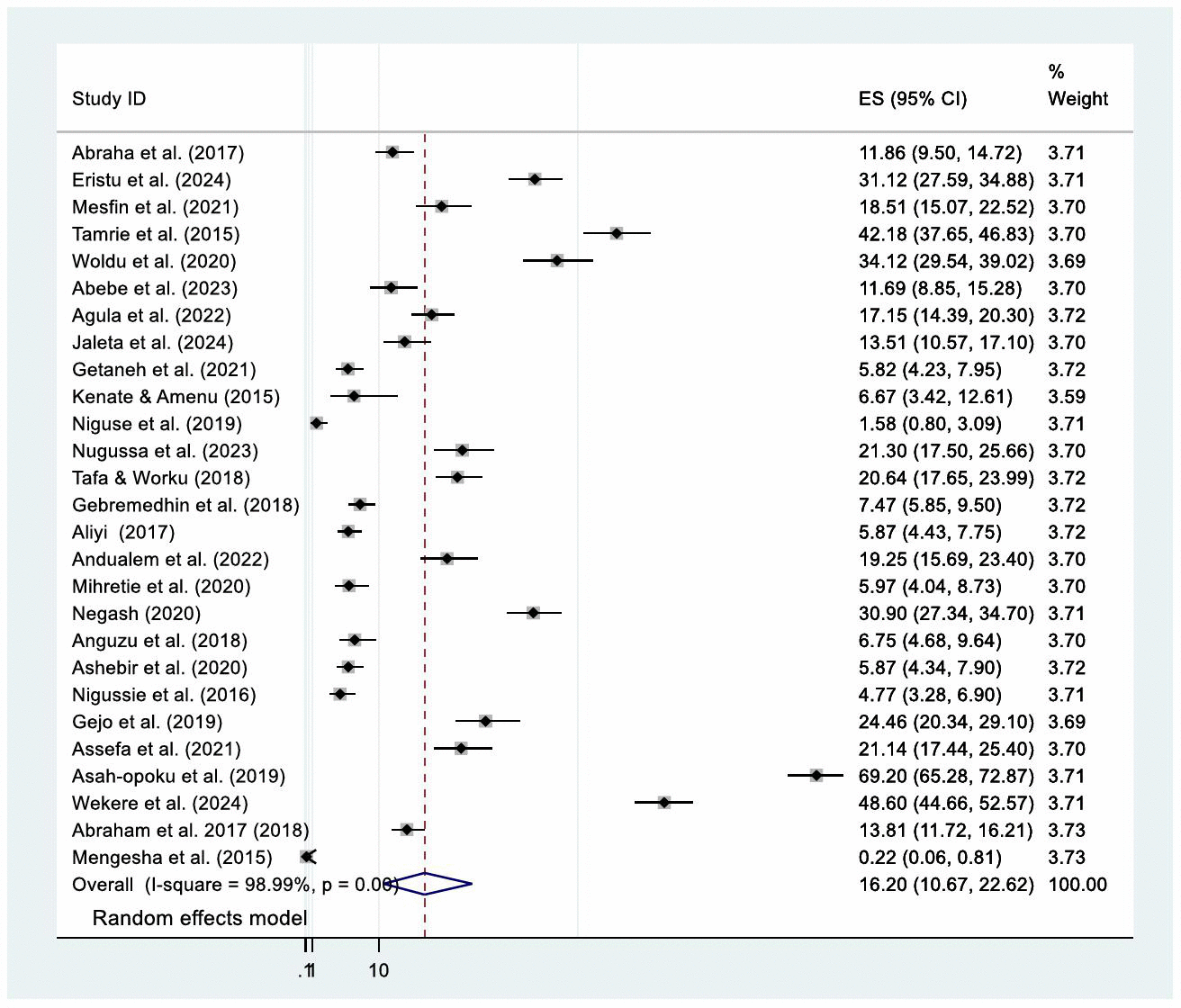
* **S6 Fig.** Pooled prevalence of EPP-I uptake in Eastern and Western Africa, 2025.(TIF)

Supplement: S6 Fig — (DOCX) [file pone.0346885.s014.docx]

*
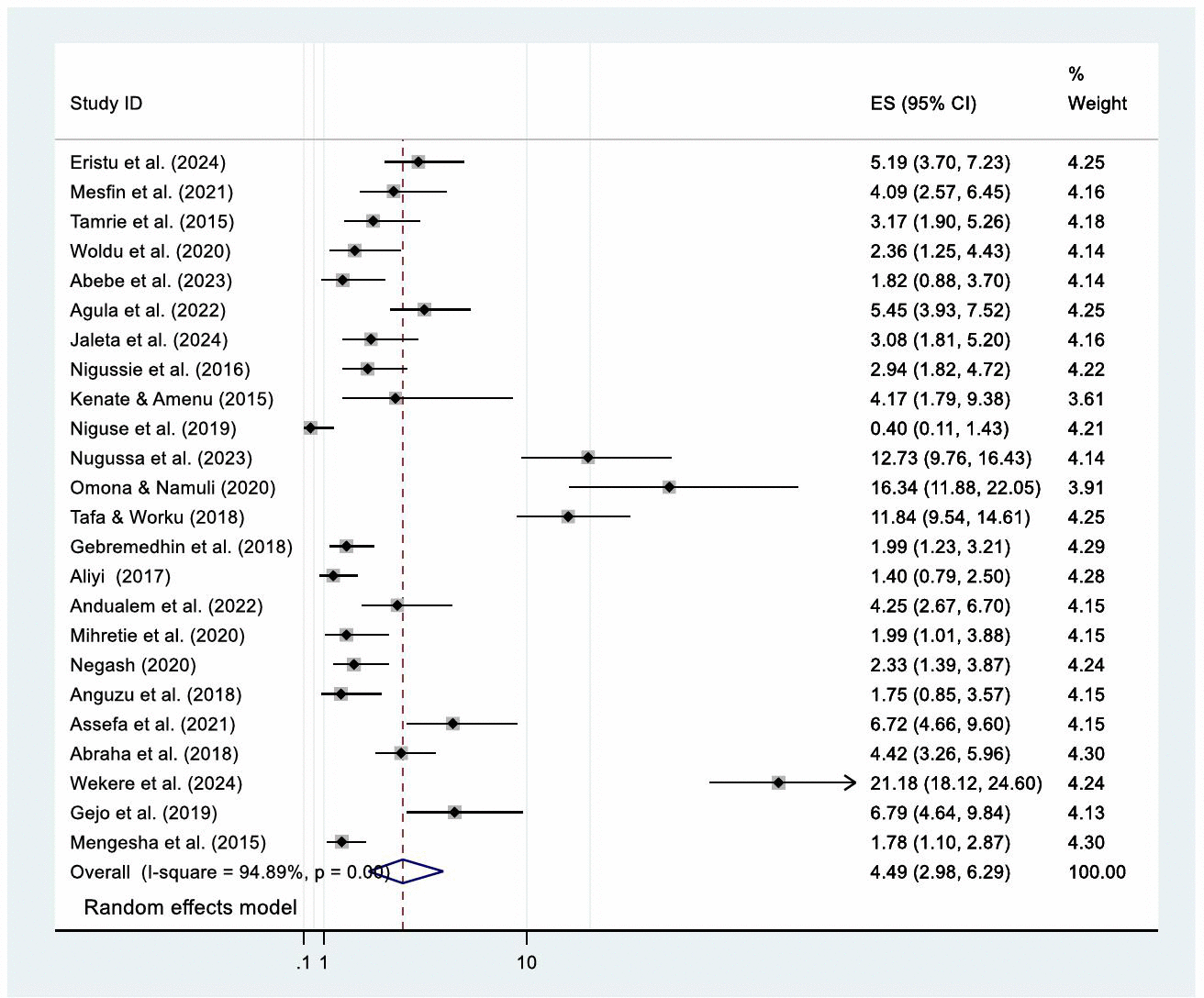
*

**S7 Fig**. Pooled prevalence of EPP-IUD uptake in Western and Eastern Africa, 2025.(TIF)

Supplement: S7 Fig — (DOCX) [file pone.0346885.s015.docx]
