# Supplementary material for: Uptake and determinants of immediate and extended postpartum long-acting reversible contraceptive use in Eastern and Western Africa: A systematic review and meta-analysis
Source: PLoS One. 2026 Apr 17;21(4):e0346885. doi: 10.1371/journal.pone.0346885 (PMC13089893; doi:10.1371/journal.pone.0346885)
Supplement: S8 Fig — (DOCX) [file pone.0346885.s016.docx]

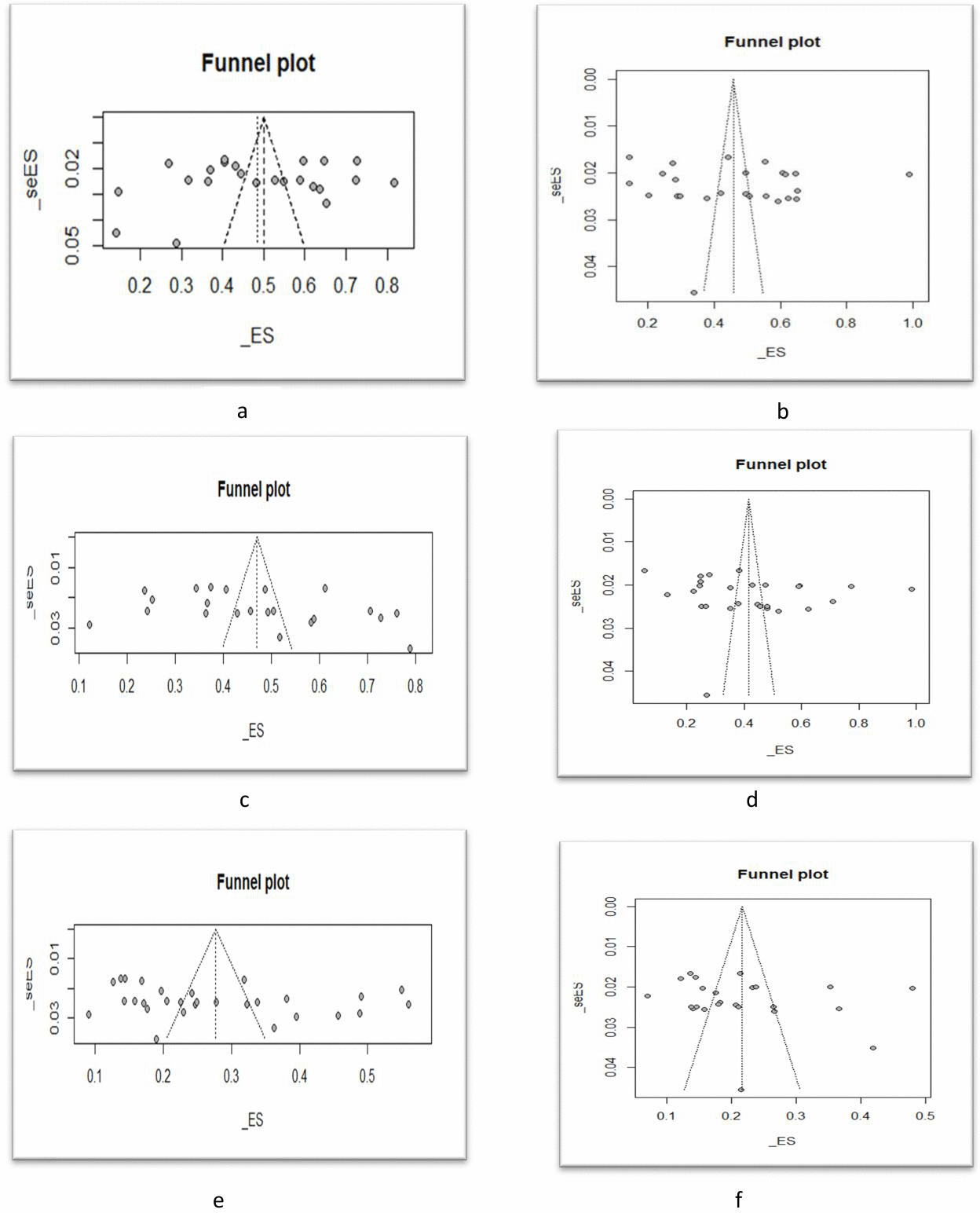


**S8 Fig.** Funnel plots assessing publication bias across immediate and early postpartum contraceptive outcomes: (a) IPP-LARC, (b) EPP-LARC, (c) IPP-I, (d) EPP-I, (e) IPP-IUD, (f) EPP -IUD.(TIF)
