# Supplementary material for: Uptake and determinants of immediate and extended postpartum long-acting reversible contraceptive use in Eastern and Western Africa: A systematic review and meta-analysis
Source: PLoS One. 2026 Apr 17;21(4):e0346885. doi: 10.1371/journal.pone.0346885 (PMC13089893; doi:10.1371/journal.pone.0346885)
Supplement: S9 Fig — Significant upward trends were observed for IPP-LARC and EPP-IUCD, while EPP-LARC, IPP-I, EPP-I, and IPP-IUCD showed no statistically significant monotonic change. [file pone.0346885.s017.docx]

*
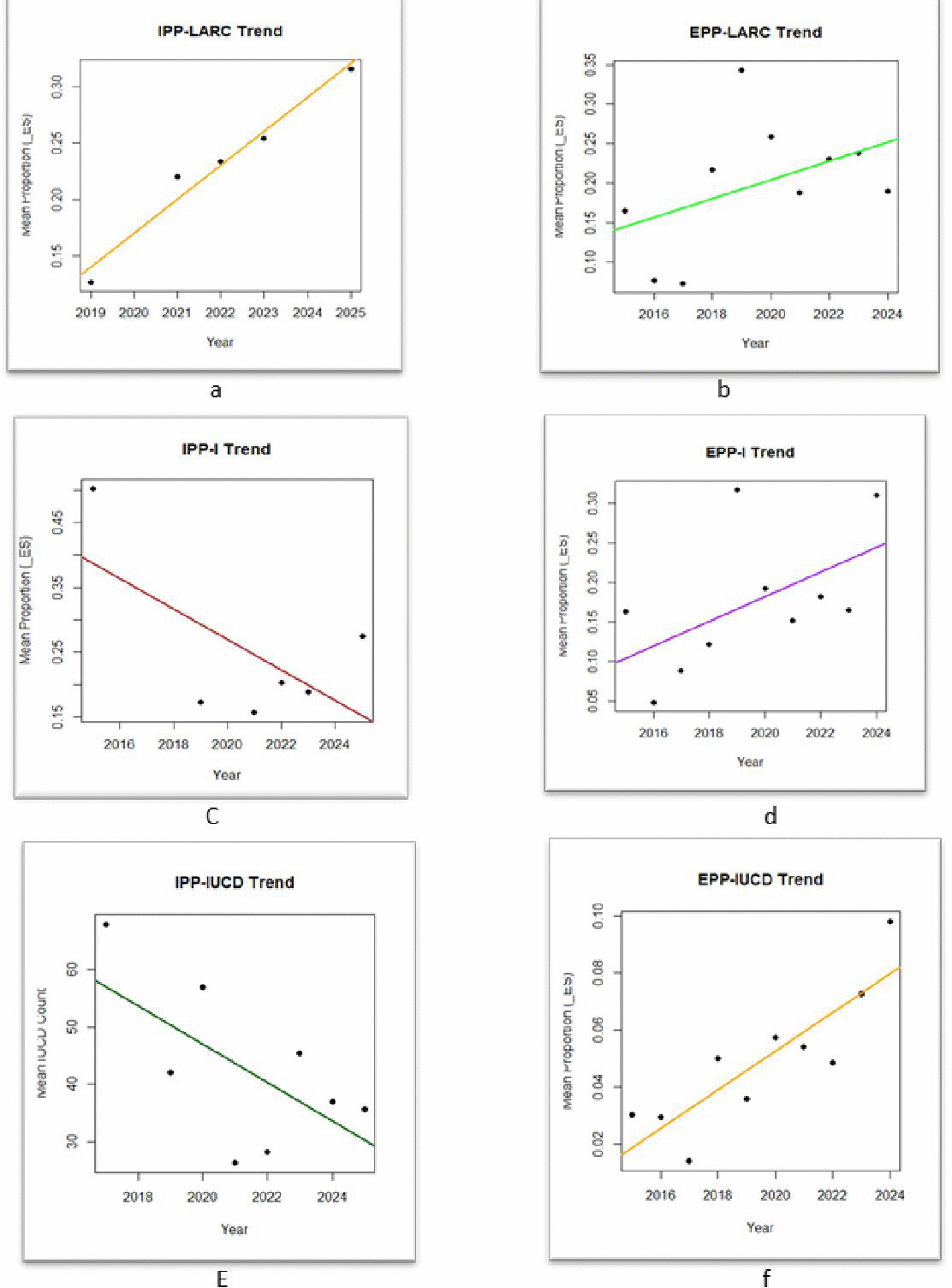
*

**S9 Fig.** Trends in postpartum contraceptive uptake across study years based on mean effect sizes: (a) IPP‑LARC, (b) EPP‑LARC, (c) IPP‑I, (d) EPP‑I, (e) IPP‑IUCD, and (f) EPP‑IUCD. (TIF) Significant upward trends were observed for IPP‑LARC and EPP‑IUCD, while EPP‑LARC, IPP‑I, EPP‑I, and IPP‑IUCD showed no statistically significant monotonic change.
